# Structure, Absolute Configuration, Antiproliferative and Phytotoxic Activities of Icetexane and Abietane Diterpenoids from *Salvia carranzae* and Chemotaxonomic Implications

**DOI:** 10.3390/molecules29061226

**Published:** 2024-03-09

**Authors:** Celia Bustos-Brito, Juan Pablo Torres-Medicis, Brenda Y. Bedolla-García, Sergio Zamudio, Teresa Ramírez-Apan, Martha Lydia Macías-Rubalcava, Leovigildo Quijano, Baldomero Esquivel

**Affiliations:** 1 Instituto de Química, Universidad Nacional Autónoma de México, Circuito Exterior, Ciudad Universitaria, Ciudad de México 04510, Mexico; jupa_001@yahoo.es (J.P.T.-M.); mtrapan@yahoo.com.mx (T.R.-A.); mmacias@iquimica.unam.mx (M.L.M.-R.); quijano@unam.mx (L.Q.); 2 Instituto de Ecología, A. C., Centro Regional del Bajío, P. O. Box 386, Pátzcuaro 61600, Mexico; brenda.bedolla@inecol.mx; 3 Independent Researcher, P. O. Box 392, Pátzcuaro 61600, Mexico; szamudioruiz@gmail.com

**Keywords:** *Salvia carranzae*, icetexane diterpenoids, abietane diterpenoids, antiproliferative activity, phytotoxicity

## Abstract

From the aerial parts of *Salvia carranzae* Zamudio and Bedolla, three new icetexane-type diterpenoids were isolated. Their structures were established through spectroscopic methods and named the following: salvicarranzanolide (**1**), 19-deoxo-salvicarranzanolide (**2**) and 19-deoxo-20-deoxy-salvicarranzanolide (**3**). In addition, the known icetexane-type diterpenoids, 6,7,11,14-tetrahydro-7-oxo-icetexone (**4**), *iso*-icetexone (**5**), 19-deoxo-*iso*-icetexone (**6**), icetexone (**7**), 19-deoxo-icetexone (**8**) and 7α-acetoxy-6,7-dihydroicetexone (**9**), were also isolated, along with the abietanes sessein (**10**) and ferruginol (**11**). α-Tocopherol was also identified. Compounds **5**, **6** and **8** were tested for their antiproliferative activity using the sulforhodamine B assay on six cancer and one normal human cell lines. Diterpenoids **5** and **6** showed noteworthy antiproliferative activity, exhibiting an IC_50_ (μM) = 0.43 ± 0.01 and 1.34 ± 0.04, respectively, for U251 (glioblastoma), an IC_50_ (μM) = 0.45 ± 0.01 and 1.29 ± 0.06 for K5621 (myelogenous leukemia), 0.84 ± 0.07 and 1.03 ± 0.10 for HCT-15 (colon cancer), and 0.73 ± 0.06 and 0.95 ± 0.09 for SKLU-1 (lung adenocarcinoma) cell lines. On the other hand, the phytotoxicity of compounds **5**–**7** and **9**–**10** was evaluated on seed germination and root growth in some weeds such as *Medicago sativa*, *Panicum miliaceum*, *Amaranthus hypochondriacus* and *Trifolium pratense* as models. While compounds **5** and **10** exhibited a moderate inhibitory effect on the root growth of *A. hypochondriacus* and *T. pratense* at 100 ppm, the diterpenoids **6**, **7** and **9** were ineffective in all the plant models. Taxonomic positions based on the chemical profiles found are also discussed.

## 1. Introduction

The genus *Salvia* L., comprising approximately 1000 species worldwide, is one of the largest genera of angiosperms [1]. The genus was organized in 1876 into four subgenera by Bentham (*Salvia*, *Leonia*, *Sclarea* and *Calosphace*) [2]; however, its diterpenoid content [3] and recent taxonomic work, including molecular phylogenetic analysis, suggest a reconsideration of this classification [4,5]. Some authors have considered the existence of additional subgenera and the taxonomic complexity of such a rich genus has led to two main proposals regarding taxonomic treatment: on the one hand, the splitting of *Salvia* into six separate genera, and on the other hand, the inclusion of the species of the related genera *Dorystaechas*, *Meriandra*, *Perovskia*, *Rosmarinus* and *Zhumeria* as part of *Salvia* [6,7]. The second proposal is based on phylogenetic analysis using two low-copy nuclear gene regions, in addition to chloroplast and nuclear ribosomal DNA, thus providing a broader definition of the genus. Recently, the analysis of phylogeny and staminal evolution of East Asian species of *Salvia* has led to the proposal of the subgenus *Glutinaria* for the inclusion of the Chinese, Japanese and Korean species. The most recent subgeneric classification of *Salvia* L., following a broader definition, comprises ten subgenera, including almost 95% of recognized species [8].

Regardless of these two trends, there is a general agreement that the subgenus *Calosphace* (Benth.) is the most diverse of the genus, comprising between 61 and 64% of the world’s species. It is also accepted that *Salvia* species developing in Mexico and Central and South America and belong mainly to this subgenus. *Salvia* is the most species-rich genus in Mexico, with approximately 312 species, representing 55% of the *Calosphace* subgenus and 30% of the world estimate [9]. On the other hand, 82% of Mexican species are endemic [10]. Due to this diversity of species and endemism, Mexico is considered the center of the origin and diversification of the subgenus *Calosphace*. The exact number of *Salvia* species in Mexico is constantly changing due to the discovery of new species. This is a consequence of the very intensive research dealing with the investigation of regional floras across the country performed in the last three decades [11,12]. Since 2007, 62 new species of the genus have been described [13,14,15,16,17,18]. *S. carranzae* Bedolla and Zamudio is a perennial herb that grows in temperate forests and was described for the first time in 2015 in the framework of the project, *Flora del Bajío y de Regiones Adyacentes*. Although it was tentatively classified in section *Fulgentes* (Epling), its taxonomic position within the subgenus *Calosphace* is uncertain, since its morphological characteristics do not exactly coincide with any of the sections proposed by Epling et al. [19,20]. In its original description, *S. carranzae* was compared with species of the *Fulgentes* section, particularly with *S. fulgens* Cav., but it differs from this and the other species of the section by its leaves with an irregularly toothed margin, the tube of its corolla missing papillae and the upper branch of its style being shorter than the lower one [19]. *S. carranzae* was also compared with species from sections *Blackea*, *Glareosae*, *Brandegeei* and *Nobiles*, but it did not completely coincide with any of them [20]. From a phytochemical point of view, section *Fulgentes* is characterized by the presence of neo-clerodane, rearranged neo-clerodane and pimarane diterpenoids, as have been described in *Salvia fulgens* [21,22,23], *S. lineata* [24] and *S. microphylla* [22,25,26,27]. 

As a result of the systematic phytochemical study of Mexican salvias, it can be stated that diterpenoids are the most characteristic constituents of the genus, and there exists a certain parallelism between the chemical composition of species and the botanical sections to which they belong, according to Epling, as in the cases of sections *Erythrostachys*, *Tomentellae* and *Scorodonia*. On the other hand, it has been possible to show that the chemical composition of *Salvia* species belonging to the subgenus *Calosphace* differs significantly from that found in species from Europe and Asia. The diterpenes most frequently found in Mexican salvias are of a neo-clerodane type or derived from this skeleton, although some abietane, icetexane, pimarane, totarane and recently labdane-type diterpenoids have also been found [28]. It is considered that these compounds may be the basis, in part, of the use of many *Salvia* species in traditional medicine in many countries. Several species of the genus *Salvia* are used as medicinal herbs in different regions of the world, for example, the Mediterranean species *S. officinalis* L. and *S. sclarea* L. have been used for centuries in different areas of Europe and the roots of *S. miltiorrhiza* are very appreciated in traditional Chinese medicine [29,30]. In Mexico, the genus is widely used for medicinal purposes and several species with similar uses are grouped in medicinal mixtures, such as *S. microphylla* Kunth, *S. coccinea* Juss. ex Murr., *S. elegans* Vahl, *S. fulgens* Cav. and *S. involucrata* Cav., which constitute the “Mirto” complex and are used to relieve stomach ailments, for example, stomachache, diarrhea, stomach cramps, colic, dysentery, stomach infections. They are even used as sedatives and muscle relaxants. On the other hand, *S. lavanduloides* Kunth and *S. longispicata* Mart. and Gal. constitute the “Cantueso” complex and are used mainly for respiratory conditions [31].

In this work, we describe the results of the phytochemical analysis of the new species, *S. carranzae*, hoping that these results will help to establish its most suitable taxonomic position and, on the other hand, to evaluate the biological activity of its isolated secondary metabolites. From the analysis of the dichloromethane extract of the aerial parts of *S. carranzae*, nine diterpenes with an icetexane skeleton were isolated, in addition to two abietanes and α-tocopherol. Some of these compounds were evaluated to establish their antiproliferative capacity against human cancer cell cultures and phytotoxicity against some weeds.

## 2. Results and Discussion

### 2.1. Characterization

**Figure 1:**
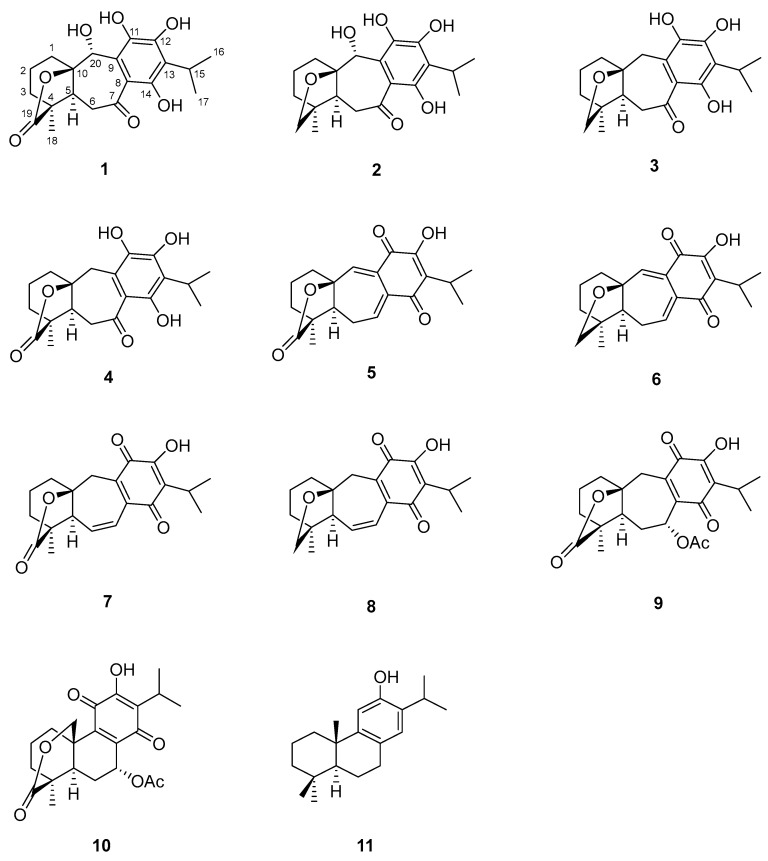
Chemical structures of **1**–**11**.

The aerial parts of *S. carranzae* yielded, after dichloromethane extraction and thorough chromatographic purification, α-tocopherol and eleven diterpenoids: three new icetexane-type (**1**–**3**), in addition to the already known icetexanes (**4**–**9**) and abietanes (**10**–**11**) (Figure 1).

Structural elucidation and identification of the isolated compounds were performed by spectroscopic methods and comparison with the literature data. 

Salvicarranzanolide (**1**) was isolated as a yellow solid; m.p. 178–180 °C. The HR-DART-MS showed a pseudomolecular ion [M + H]^+^ at a *m*/*z* 377.1586 (calculated for C_20_H_24_O_7_ + H, 377.1600), establishing a molecular formula, C_20_H_24_O_7_, indicating a high degree of unsaturation (Ω = 9). In the ^13^C-NMR of **1** (Table 1), in addition to the characteristic signals of an icetexone-type diterpenoid derivative at δ_C_ 178.8 and 87.5, due to carbons of the γ-lactone carbonyl (C-19, C-10) and C-18 methyl group at 16.9, signals for a fully substituted benzene ring at δ_C_ 109.8 (C-8), 114.1 (C-9), 136.9 (C-11), 151.9 (C-12), 121.2 (C-13) and 158.8 (C-14) were also observed [28]. One of the substituents of the benzene ring was identified as the typical *iso*-propyl group at C-13, since the expected signals for this moiety were observed at δ_C_ 24.5 (C-15), 20.1 (C-16) and 19.8 (C-17). A signal at δ_C_ 202.7 was ascribed to a conjugated ketone carbonyl located at C-7. The IR spectrum of salvicarranzanolide (**1**) showed bands due to hydroxy groups (3593, 3500 and 3352 cm^−1^), indicating that the other substituents of the aromatic ring must have been hydroxy groups. The IR spectrum also showed the γ-lactone and conjugated ketone carbonyl bands at 1776 and 1601 cm^−1^, respectively, confirming the presence of these groups in **1**. Similar functionalities were observed for 6,7,11,14-tetrahydro-7-oxo-icetexone (**4**) an icetexane-type diterpenoid previously isolated from *Salvia ballotiflora* [28] and also present in *S. carranzae*.

**Figure 2:**
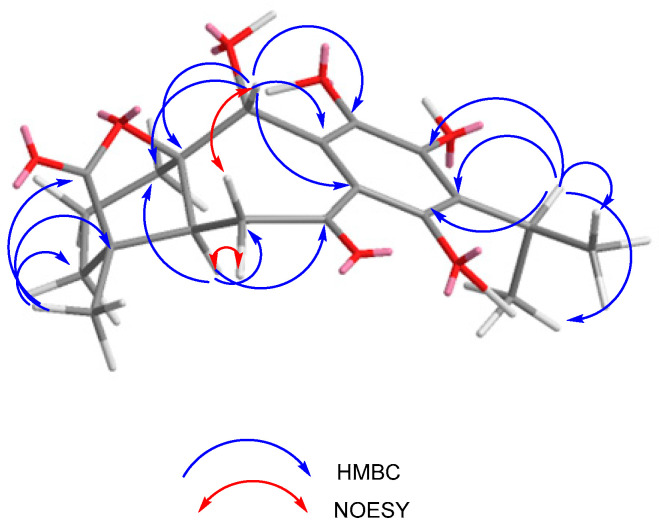
Selected HMBC and NOESY correlations for compound **1**.

**Figure 3:**
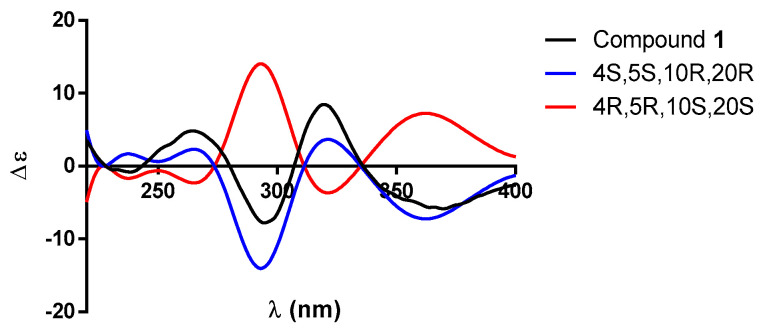
Comparison between the experimental and calculated ECD spectra of **1** (black line) and the enantiomers 4*S*,5*S*,10*R*,20*R* (blue line) and 4*R*,5*R*,10*S*,20*S* (red line).

In the ^1^H-NMR spectrum of **1** (Table 1), signals for an ABX system were observed at δ_H_ 2.72 (dd, *J* = 17.5, 12.4 Hz), 2.80 (dd, *J* = 17.5, 1.5 Hz) and 2.11 (dd, *J* = 12.4, 1.5 Hz). The chemical shift and coupling constants of the AB methylene proton signals indicate its vicinity to a carbonyl group and were ascribed to the C-6 methylene hydrogen atoms. Therefore, the X part of this system was ascribed to the axially oriented H-5. Another relevant signal in the ^1^H-NMR spectrum of **1** is a singlet at δ_H_ 5.44 which was assigned to the hydrogen atom at the C-20 position, geminal to a hydroxy group. A singlet at δ_H_ 12.76 was assigned to a hydrogen-bonded hydroxy group between the C-7 carbonyl group and the hydrogen atom of the hydroxyl group at the aromatic C-14. Based on the above evidence, the structure of compound **1** was established as the C-20 hydroxy derivative of compound **4**. The HMBC spectrum of salvicarranzanolide (**1**) supports the previous considerations since the expected correlation cross peaks were observed as depicted in Figure 2. The relative configuration of salvicarranzanolide (**1**) was established according to coupling constants and nOe interactions observed in the NOESY spectrum (Figure 2). The nOe correlation observed between H-20 and H-6*β* suggested an α-orientation for the hydroxy group at C-20. To establish the absolute configuration of compound **1**, the excitation energy (nm) and rotatory strength (R) in dipole velocity (Rvel) for the 4*S*,5*S*,10*R*,20*R* isomer and its enantiomer were calculated using TD-DFT and simulated into ECD curves (Figure 3). The experimental ECD spectrum for **1** displayed two positive Cotton effects at 265 and 320 nm, two negatives at 295 and 365 nm, and was in good agreement with the calculated data for the 4*S*,5*S*,10*R*,20*R* enantiomer depicted in **1**. 

**Figure 4:**
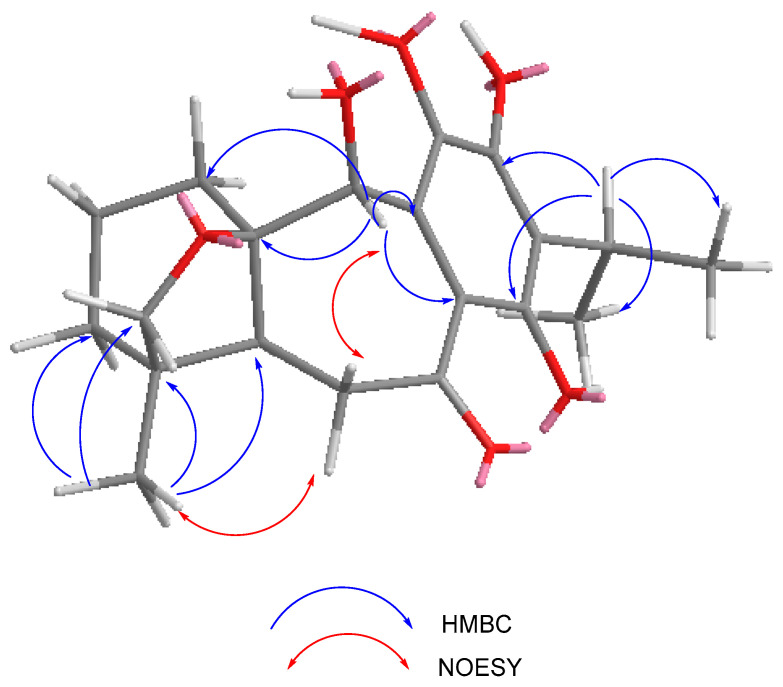
Selected HMBC and NOESY correlations for **2**.

**Figure 5:**
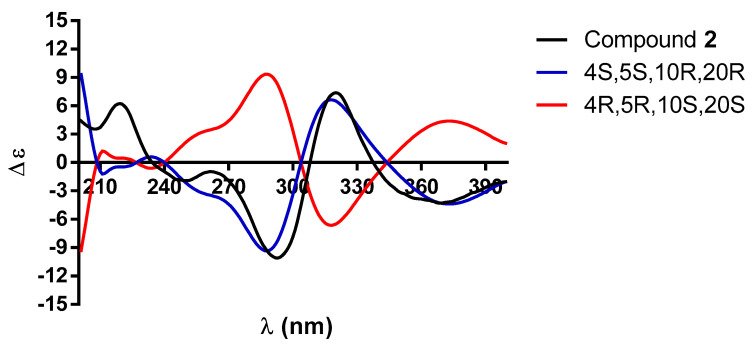
Comparison between the experimental and calculated ECD spectra of **2** (black line) and the enantiomers 4*S*,5*S*,10*R*,20*R* (blue line) and 4*R*,5*R*,10*S*,20*S* (red line).

19-Deoxo-salvicarranzanolide (**2**) was isolated as a yellow powder; m.p. 140–142 °C; [α]_D_-160 (c 0.0006, MeOH). The HR-DART-MS indicated a C_20_H_26_O_6_ molecular formula. Its IR spectrum showed bands at 3591, 3501 and 3319 cm^−1^, ascribed to hydroxyl groups, and 1599 cm^−1^, assigned to a conjugated ketone carbonyl. The ^13^C-NMR spectrum (Table 1) displayed signals for 20 carbons accounting for three methyls, five methylenes, three methines and nine quaternary carbons, which include two quaternary sp^3^, one carbonyl and six aromatic carbons, according to HMBC and HSQC experiments. The NMR data of compound **2** (Table 1) are similar to those obtained for salvicarranzanolide (**1**), with the most significant differences being the absence of a γ-lactone carbonyl group in the ^13^C-NMR of **2** and the presence of an AB system at δ_H_ 3.90 (d, *J* = 7.9 Hz) and 3.72 (dd, *J* = 7.9, 2.1 Hz), ascribed to the C-19 methylene hydrogen atoms. The C-19 *pro*-*R* hydrogen atom showed an additional long-range coupling with H-3α. The previous considerations were supported by the correlation cross peaks observed in the HMBC spectrum (Figure 4). The relative stereochemistry of compound **2** was established by the analysis of coupling constants and nOe observed in the NOESY spectrum. A cross peak of correlation between H-20 and H-6*β* led us to propose an *α*-orientation for the hydroxyl group at C-20, as in compound **1**. The absolute configuration of compound **2** was established as 4*S*,5*S*,10*R*,20*R* based on the results obtained from the comparison between the theoretical (blue and red lines) and experimental ECD curves (black line, Figure 5), which displayed a negative Cotton effect at 295 nm and a positive effect at 320 nm. 

Compound **3** was obtained as a yellow powder. Its molecular formula was determined to be C_20_H_26_O_5_ from the [M + H]^+^ pseudomolecular ion observed at a *m*/*z* 347.1853 (calculated 347.1858) in HR-DART-MS, indicating a high degree of unsaturation (Ω = 8). The IR spectrum showed bands ascribed to hydroxyl groups (3317 cm^−1^), a conjugated ketone carbonyl group (1597 cm^−1^) and aromatic double bonds (1570 cm^−1^). Mass and IR spectra, together with NMR data (Table 1), led us to establish the structure depicted in **3** for this novel icetexane-type diterpenoid. The ^13^C NMR signals for a fully substituted benzene ring were observed at δ_C_ 126.3 (C-8), 113.6 (C-9), 136.4 (C-11), 154.9 (C-12), 120.3 (C-13) and 160.5 (C-14). The chemical shifts of these signals indicated the presence of hydroxyl groups at the C-11, C-12 and C-14 positions. Signals of an *iso*-propyl group at the C-13 position were also observed at δ_C_ 25.7 (C-15), 20.5 (C-16) and 20.6 (C-17). The signal for the C-7 keto carbonyl was observed at δ_C_ 208.1. These facts indicated that compound **3** possesses a similar fully substituted aromatic ring conjugated with a ketone group at C-7, as diterpenoids **1** and **2**.

**Figure 6:**
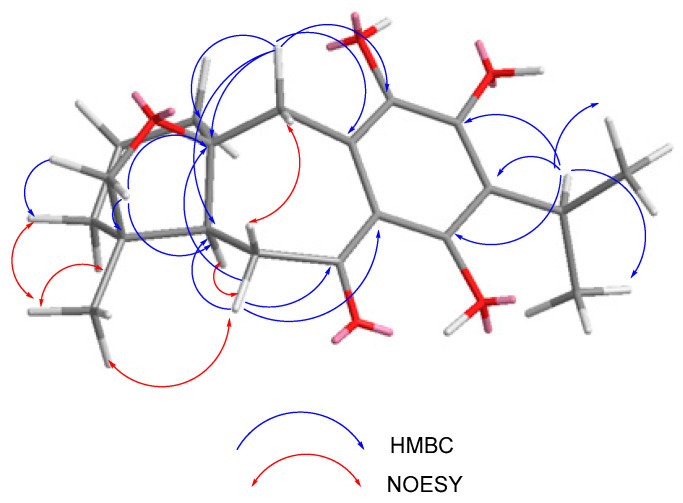
Selected HMBC and NOESY correlations for compound **3**.

**Figure 7:**
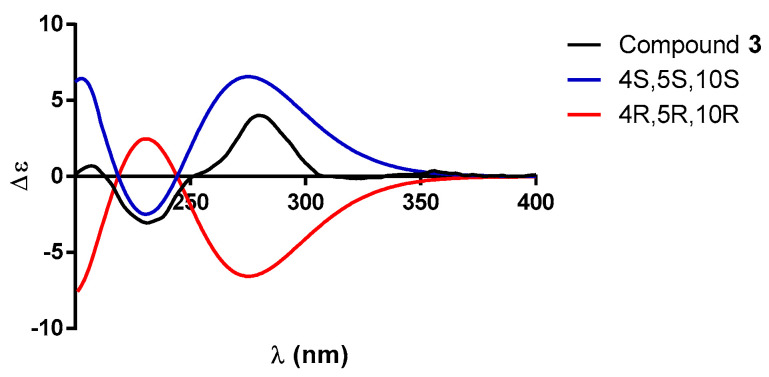
Comparison between the experimental and calculated ECD spectra of **3** (black line) and the enantiomers 4*S*,5*S*,10*S* (blue line) and 4*R*,5*R*,10*R* (red line).

In the ^1^H NMR spectrum of **3** (Table 1), the signals for an AB system at δ_H_ 3.44 (d, *J* = 13.6 Hz) and 2.84 (d, *J* = 13.6 Hz) were observed and attributed to the C-20 methylene hydrogen atoms, characteristic of an icetexane-type diterpenoid. Compound **3**, therefore, is devoid of the hydroxy group at the C-20 position present in compounds **1** and **2**. Another AB system was observed at δ_H_ 3.86 (d, *J* = 7.7 Hz) and 3.72 (dd, *J* = 7.7 and 2.2 Hz) and assigned to the C-19 methylene hydrogen atoms. The *pro*-R hydrogen of this group exhibited a long-range coupling of 2.2 Hz with H-3α in agreement with a *β* orientation for the ether linkage between C-4 and C-10. Compound **3** could be considered to be the C-20 deoxy derivative of **2** and named 20-deoxy-19-deoxo-salvicarranzanolide (**3**). The relative and absolute configuration of **3** were established by nOe cross peaks of correlation in the NOESY spectrum (Figure 6) and by TD-DFT calculation of the ECD (Figure 7), respectively. The absolute configuration of compound **3** was established as 4*S*,5*S*,10*S*. 

**Scheme 1:**
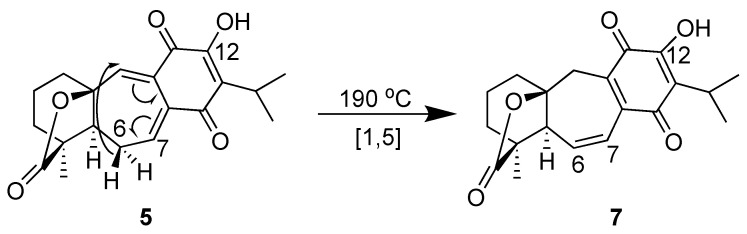
Thermically induced transposition of *iso*-icetexone (**5**) to icetexone (**7**) through a [1,5] H sigmatropic reaction.

The icetexane **5**, named *iso*-icetexone, was recently described from a population of *Salvia uliginosa* Benth (section *Uliginosae*) collected in Brazil, together with icetexone (**7**) and compound **9** [29]. In the ^1^H NMR spectrum of **5**, obtained in CDCl_3_, a complex signal is observed for the hydrogen of the C-7 position at δ 7.37 ppm, which was described as a multiplet by the authors who originally isolated this compound [29]. The complexity of this signal suggests the existence of conformers in the CDCl_3_ solution. Inspection of a Dreiding model of **5** revealed that it is a rigid molecule and that the possibility of conformers affecting the appearance of its H-7 may be due to the movement of the bonds of the molecule around C-6. When the ^1^H NMR spectrum of **5** was obtained in CD_2_Cl_2_ as a solvent, this complex signal was observed as a clear double doublet as expected at δ 7.35. The complete ^1^H and ^13^C data for *iso*-icetexone (**5**) recorded in CD_2_Cl_2_ are included in the experimental section. Additionally, when compound **5** was placed on the heating block of the Fisher-Johns apparatus to obtain its melting point, a color change from yellow to orange began to be observed around 160 degrees Celsius, which was completed when the temperature reached 190 degrees. When the ^1^H NMR spectrum of the orange crystals recovered from the Fisher-Johns was obtained, the *iso*-icetexone (**5**) signals were no longer observed and the spectrum corresponds to icetexone (**7**), indicating isomerization of **5** to **7** under heating conditions. This transformation could be considered as a [1,5] H sigmatropic rearrangement, which is allowed under thermal conditions, as indicated in Scheme 1. This isomerization takes place slowly, even at room temperature, as shown in a series of ^1^H NMR spectra taken over several months (see Supplementary Materials Figure S29). After each determination, the solvent was evaporated, and the dried sample was left at room temperature until the next one. As can be observed, over 10 months, the signals corresponding to the vinylic hydrogen atoms H-6 and H-7 at δ 6.43 and 6.86, respectively, and the hydrogen of -OH at C-12 (δ 7.11) corresponding to icetexone (**7**), appeared and increased, while the *iso*-icetexone signals decreased. A similar behavior was observed for compound **6**, since when it was heating in the Fisher-Johns apparatus, it was transformed into the diterpenoid **8**. Isomerization started at 175 °C and completed at 185 °C. A similar mechanism could be proposed for this rearrangement.

Compounds **4** and **6**–**9** have been previously isolated from different populations of *S. ballotiflora* Benth (section *Tomentellae*). Compounds 6,7,11,14-tetrahydro-7-oxo-icetexone (**4**) and 7α-acetoxy-6,7-dihydroicetexone (**9**) were isolated from a population collected in the State of Nuevo Leon (Mexico). Both compounds showed significant antiproliferative activity in a panel of six human cancer cell lines [28]. Samples collected in the Municipality of Guadalcázar in the State of San Luis Potosi (Mexico) led to the isolation of 19-deoxo-*iso*-icetexone (**6**), icetexone (**7**) and 19-deoxo-icetexone (**8**). Compound **6** proved to be very active against Hela cells approaching *cis*-platin, while compounds **7** and **8** showed inhibition of the production of NO and decreased the concentration of *pro*-inflammatory cytokine in macrophages, and compound **8** showed antidiarrheal activity in a rodent model [30]. Even though icetexone (**7**) was the first isolated icetexane [9(10 → 20)-*abeo*-abietane] diterpenoid originally isolated from *Salvia ballotiflora* several years ago, its absolute configuration and the full assignments of its ^1^H and ^13^C NMR were only recently published [31]. Compounds **5** and **7** exhibited potent antichemotactic and leishmanicidal activities [29]. Ferruginol (**11**) was obtained several years ago from *Podocarpus ferruginea* (Podocarpaceae) [32] but is also present in plants of the Cupressaceae, Verbenaceae and Lamiaceae families and has shown a plethora of biological activities [33]. 

Although the diterpenoid content found for *S. carranzae* resembles that found for *Salvia ballotiflora* [28,31], it is strongly related to that described for the Mexican species of section *Erythrostachys* [20], chemically studied up to now for the following species: *S. sessei*, *S. pubescens* and *S. regla*. Sessein (**10**), isolated from *S. carranzae*, is a diterpene lactone originally isolated from *S. sessei* [34,35] and described in two populations of *S. regla* [36,37]. No other sources of sessein (**10**) have been described up to now. On the other hand, icetexone (**7**), as well as 19-deoxo-icetexone derivatives, such as **2**–**3**, **6** and **8**, found in *S. carranzae*, have also been isolated from *S. pubescens* [38], thus reinforcing the chemical relationship between *S. carranzae* and the species of section *Erythrostachys*. *S. carranzae* shares with the members of section *Erythrostachys* ovate-acuminate leaves and flowers with the following characteristics: glabrous to rarely pilose style, red calyx and corolla, the latter ± 5 cm long with an infundibular, epapillated, non-invaginate tube and subequal lips, with the lower one narrow. In its original description, it was compared with species of the *Fulgentes* section, particularly with *S. fulgens* Cav., but it differs in a very marked way in its type of diterpenoids produced. *S. carranzae* was also compared with species from sections *Blakea*, *Glareosae*, *Brandegeei* and *Nobiles*, but it does not completely coincide with any of them [19].

*S*. *carranzae* has not yet been included in any molecular phylogenetic analyses, and thus, its exact relationship is not known. We suspect that it belongs to the basal *Erythrostachys* clade, which also includes species of sections *Blakea* and *Glareosae*. Like *S*. *carranzae*, members of this clade have styles with a longer lower branch than the upper branch, a characteristic of the species already mentioned by the present authors [19]. It is necessary to carry out chemical studies in the *Blackea* and *Glareosae* sections, to compare the diterpenoid content of representative species of both sections with that of *S. carranzae*. The results described in this work support the inclusion of *S. carranzae* in the section *Erythrostachys*; however, a definitive solution to the taxonomic position of this new species could be found when phylogenetic studies are carried out. It is pertinent to clarify that recent works have shown that most of the sections proposed by Epling et al. for the *Calosphace* subgenus do not phylogenetically represent related groups [39,40].

### 2.2. Biological Activity

#### 2.2.1. Antiproliferative Activity

The family Lamiaceae is one of the richest sources of diterpenoids, with some of them exhibited promising anticancer activity [41]. Diterpenes of the abietane and icetexane type have demonstrated interesting antiproliferative activities, such as anastomosin from *Salvia anastomosans* and *S. ballotiflora*, as well as compound **9** (6,7-dihydro-7α-acetoxy icetexone) from *S. ballotiflora* [28], which is also present in *S. carranzae*. These results motivated us to evaluate the major products **5**, **6** and **8**, isolated from *S. carranzae* in a panel of human cancer cell lines. The primary screening for the inhibition of cancer cell development by compounds **5**, **6** and **8** is shown in Table S1 (Supplementary Materials). At a concentration of 25 μM, compounds **5** and **6** were shown to be highly cytotoxic across the entire panel, which included a normal cell line derived from a monkey kidney (COS-7). When tested at a concentration of 1 μM, compound **5** exhibited strong inhibition of cell lines U251, K562, HCT-15 and SKLU-1 and showed moderate toxicity for healthy monkey kidney cells (COS-7). On the other hand, product **6** demonstrated high activity at 1 μM against K562 and SKLU-1 cell lines, with minimal cytotoxicity against COS-7 cells. Compound **8**, however, did not show cytotoxicity in the entire panel.

Table 2 shows the results of the IC_50_ determination of compounds **5** and **6** against cell lines U251, K562, HCT-15, SKLU-1 and COS-7, compared with adriamycin, as a positive control. Compound **5** was shown to be active in all cell lines tested, although its selectivity index, calculated using the COS-7 line as a normal cell line, indicates low selectivity. It is interesting to note that the two active diterpenes **5** and **6** have a 1,4-diene system between carbons C-7 to C-20, conferring a quinoid character to these products. Similar functionality can be found in anastomosin, an icetexane-type diterpene whose antiproliferative activity approaches that of adriamycin and was originally isolated from *Salvia anastomosans* and recently, from *S. ballotiflora* [28].

#### 2.2.2. Phytotoxic Activity

Plants of the genus *Salvia* in Mexico are dominant species in their habitat, with evident allelopathic effects, making them an ideal source for the extraction of secondary metabolites with potentially useful phytotoxic effects. Recently, the phytotoxic effect of exudates from several *Salvia* species was studied, showing strong inhibitory effects on the germination of *Papaver rhoeas* and *Avena sativa* [42]. These facts added to the need for new herbicides for sustainable agricultural production in Mexico, which motivated us to evaluate the phytotoxic effect of some of the isolated products of *S. carranzae* on the germination and elongation of the roots of four species of plants considered in Mexico as weeds: the dicotyledons *Trifolium pratense*, *Medicago sativa* and *Amaranthus hypochondriacus* and the monocotyledon *Panicum miliaceum*. Compounds **5**–**7**, **9** and **10** were tested for their inhibitory effects on the germination and elongation of the roots on the previously mentioned model plants.

While compound **5** showed moderate inhibition on *A*. *hypochondriacus* root elongation, with a 54.82 ± 0.05% of inhibition at 100 mg/mL, compound **10** showed weak inhibition on *A. hypochondriacus* (35.54 ± 0.11) and *P*. *miliaceum* (35.20 ± 0.11). None of the tested products showed activity in the inhibition of the germination of the plants used in this bioassay (Table S2, Supplementary Materials). 

## 3. Materials and Methods

### 3.1. General Experimental Procedure

Melting points were measured using a Fisher-Johns apparatus (Fisher Scientific Company, Pittsburgh, PA, USA) and are uncorrected. Optical rotations were measured on a PerkinElmer 323 polarimeter (PerkinElmer Inc., London, UK). Ultraviolet absorptions were recorded on a Shimadzu UV 160U spectrophotometer (Kyoto, Japan). ECD spectra were recorded on a JASCO-1500 polarimeter (JASCO Inc., Easton, MD, USA) in MeOH or CHCl_3_. 1D and 2D NMR experiments were performed on a Bruker Advance III spectrometer (Bruker Corporation, Billerica, MA, USA) at 700 MHz for ^1^H and 175 MHz for ^13^C. CDCl_3_, CD_2_Cl_2_ or CD_3_OD were used as solvents as indicated, and chemical shifts were referred to a residual solvent (CHCl_3_:δ_H_ = 7.26, δ_C_ = 77.16; CH_2_Cl_2_:δ_H_ = 5.32, δ_C_ = 54; CH_3_OH:δ_H_ = 3.31, δ_C_ = 49). IR spectra were obtained on an FT-IR NICOLET IS-50 spectrometer (Thermo Fisher Scientific Inc., Waltham, MA, USA). HR-DART-MS was determined on an AccuTOF JMS-T100LC mass spectrometer (Jeol Ltd., Tokyo, Japan). Silica gel with a 230–400 Macherey-Nagel mesh (Düren, Germany), precoated TLC plates (SIL G-100 UV254; Macherey-Nagel, Düren, Germany) and Sephadex LH-20 (Pharmacia Biotech AB, Uppsala, Sweden) were used for column chromatography. GC–MS was performed using an Agilent Technologies (7890B) gas chromatograph equipped with a mass detector (Agilent 5977A; Agilent, Santa Clara, CA, USA). 

### 3.2. Plant Material

*S. carranzae* was collected in the Municipality of Xilitla, San Luis Potosí, Mexico, in October 2018. Plant material was identified by Sergio Zamudio and Brenda Y. Bedolla-Garcia and deposited in the IEB herbarium of the Instituto de Ecología, A. C., Centro Regional del Bajío (Voucher IEB-266883).

### 3.3. Extraction and Isolation

Dried and powdered leaves of *S. carranzae* (300 g) were extracted by percolation with CH_2_Cl_2_ (1.5 L). The solvent was eliminated by distillation and dried by reduced pressure to yield 16.0 g of residue. The crude extract was subjected to column chromatography (CC) on silica gel using petrol/EtOAc (85:15) as the eluent to obtain 62 primary fractions (225 mL each). Compound **11** and α-tocopherol were identified by GC–MS from fraction 2. 

Compound **8** (40.0 mg) was obtained as orange crystals from fraction 5 (237.7 mg), and compound **6** (498.0 mg) was identified from fraction 6 (729.9 mg) as yellow powder. Fraction 17 (338.2 mg) was separated by column chromatography on silica gel, using a mixture of CH_2_Cl_2_/EtOAc/MeOH/H_2_O (95:4:0.6:0.4) to yield compounds **5** (40.1 mg) and **7** (56.4 mg) as yellow and red crystals, respectively. Fractions 27–42 were combined according to their chromatographic profiles to obtain 450.4 mg and further fractionated on a Sephadex LH-20 column, using MeOH as the eluent, to obtain 27 eluates (4 mL each), which were combined into five major fractions (A–E) according to their chromatographic profile. Fraction B (140.3 mg) was purified by CC on silica gel using petrol/EtOAc/MeOH/H_2_O (60:36:3:1) to obtain 27 eluates (25 mL each), which were combined into four major fractions (BA–BD) according to their chromatographic profile. Fraction BB (18.1 mg) was purified by preparative TLC using petrol/EtOAc/MeOH/H_2_O (67:30:2:1) as the mobile phase to give compound **1** (5.2 mg). Fraction BC (24.8 mg) was purified by preparative TLC using petrol/EtOAc/MeOH (77:19:4) as the mobile phase to give compound **2** (7.5 mg). Fraction BD (26.8 mg) was subjected to CC using petrol/EtOAc (100:0–0:100) to obtain 34 eluates (20 mL each), which were combined into three major fractions (BDA–BDC). Fraction BDB (15.3 mg) was subjected to preparative TLC using CH_2_Cl_2_/petrol/EtOAc/MeOH) (85:13.5:1.0:0.5) as the mobile phase to give compounds **9** (6.7 mg) and **10** (5.9 mg) as yellow powders. 

Primary fractions 43–47 were combined according to their chromatographic profiles (463.9mg) and further fractionated over a Sephadex LH-20 column, using CH_2_Cl_2_/MeOH (8:2) as the eluent, to obtain 18 eluates. Fractions 10–11 were combined, obtaining a yellow powder (21 mg), which was subjected to preparative TLC using CH_2_Cl_2_/acetone (9:1) to obtain compounds **3** (7.1 mg) and **4** (8.2 mg).

Compound **1**, Yellow powder; m.p. 178–180 °C; [α]_D_-287 (c 0.001, MeOH); UV (MeOH) λ_max_ (log ε) 214 (3.99), 243 (3.64), 300 (3.85), 348 (3.62), 369 (3.69) nm; IR (CHCl_3_) ν_max_ 3593, 3500, 3352, 2962, 2877, 1776, 1601, 1422, 1323, 1289, 1169, 1093, 935, 903 cm^−1^; ^1^H and ^13^C NMR (CDCl_3_) (see Table 1); HR-DART-MS *m*/*z* 377.1586 [M + H]^+^ (calculated for C_20_H_25_O_7_, 377.1600).
Compound **2**, Yellow powder; m.p. 140–142 °C; [α]_D_-160 (c 0.0006, MeOH); UV (MeOH) λ_max_ (log ε) 215 (4.12), 244 (3.71), 302 (3.83), 367 (3.63) nm; IR (CHCl_3_) ν_max_ 3591, 3501, 3319, 2960, 1936, 2874, 1775, 1711, 1599, 1423, 1327, 1289, 1168, 1118, 1019, 919, 193 cm^−1^; ^1^H and ^13^C NMR (CDCl_3_) (see Table 1); HR-DART-MS *m*/*z* 363.1808 [M + H]^+^ (calculated for C_20_H_27_O_6_, 363.1804).
Compound **3**, Yellow powder; m.p. 285–290 °C; [α]_D_-89 (c 0.0021, MeOH); UV (MeOH) λ_max_ (log ε) 208 (3.78), 242 (3.12), 295 (3.63), 352 (2.94) nm; IR (ATR) ν_max_ 3317, 3083, 2933, 2870, 1597, 1570, 1462, 1379, 1319, 1245, 1162, 1146, 1119, 1013, 994, 969, 901, 807, 189, 698, 641, 596, 567, 533, 498, 422 cm^−1^; ^1^H and ^13^C NMR (MeOD) (see Table 1); HR-DART-MS *m*/*z* 347.1853 [M + H]^+^ (calculated for C_20_H_27_O_5_, 347.1858).
Compound **5**, Yellow powder; m.p. 275–276 °C; [α]_D_ +352 (c 0.0013, MeOH); UV (MeOH) λ_max_ (log ε) 207 (4.07), 277 (3.91), 313 (3.92) nm; IR (CHCl_3_) ν_max_ 3383, 2965, 2938, 2879, 1775, 1670, 1642, 1614, 1397, 1382, 1336, 1276, 1193, 1117, 1018, 922 cm^−1^; ^1^H NMR (CD_2_Cl_2_, 700 MHz) δ 2.11 (1H, dd, *J* = 13.2, 5.9, H-1a), 1.50 (1H, m, H-1b), 1.89 (1H, dt, *J* = 11.7, 5.8, H-2a), 1.78 (1H, m, H-2b), 1.73 (1H, m, H-3a), 1.54 (1H, m, H-3b), 2.08 (1H, d, *J* = 14.0, H-5), 2.67 (1H, dd, *J* = 14.0, 9.5, H-6a), 2.02 (1H, dt, *J* = 14.0, 5.2, H-6b), 7.35 (1H, dd, *J* = 9.5, 5.2, H-7), 3.51 (1H, hep, *J* = 7.1, H-15), 1.25 (3H, d, *J* = 7.2, H-16), 1.24 (3H, d, *J* = 7.2, H-17), 1.21 (3H, s, H-18), 7.24 (1H, s, H-20), 7.58 (1H, s, 12-OH; ^13^C NMR (CD_2_Cl_2_, 700 MHz) δ 34.8 (CH_2_-1), 20.1 (CH_2_-2), 35.8 (CH_2_-3), 48.4 (C-4), 57.7 (CH-5), 25.8 (CH_2_-6), 143.2 (CH-7), 129.0 (C-8), 132.9 (C-9), 86.0 (C-10), 182.7 (C-11), 155.1 (C-12), 132.7 (C-13), 184.1 (C-14), 25.7 (CH-15), 19.6 (CH_3_-16), 19.8 (CH_3_-17), 17.6 (CH_3_-18), 178.0 (C-19), 140.6 (CH-20). HR-DART-MS *m*/*z* 343.1548 [M + H]^+^ (calculated for C_20_H_23_O_5_, 343.1545).

### 3.4. CG-MS Analysis

For GC–MS, 1 μL of the sample was dissolved in CH_2_Cl_2_ and injected into an Agilent Technologies (7890B) gas chromatograph equipped with a mass detector (Agilent 5977A, Agilent Technologies, Santa Clara, CA, USA), which operated using helium as a carrying gas, with a flow of 1 mL min^−1^, with a splitless injection at a temperature of 260 °C in an HP5 MS non-polar capillary column (Agilent Technologies, Santa Clara, CA, USA) (30 m × 0.25 mm I.D. × 0.25 μm film), under the following conditions: initial temperature of 40 °C, followed by an 8 °C min^−1^ ramp in order to reach a temperature of 300 °C during 6.5 min. The mass spectrometer (Agilent 5977A; Agilent Techologies, Santa Clara, CA, USA) operated at a flow of 1 mL min^−1^, with an ionization voltage of 70 eV, at an interface temperature of 230 °C, in SCAN mode and at a mass interval of 30–700 mz^−1^. The mass spectra obtained were compared with the spectra from the database NIST version 14.

### 3.5. Computational Methods

3D models for compounds **1**–**3** were built and their geometry was optimized using a semiempirical method (PM3), as implemented in Spartan’10. Conformational analysis was performed using the same software and force field. All conformers were filtered and checked for redundancy. Subsequently, the conformers were minimized and optimized, and thermochemical properties were obtained with Gaussian 09 using a DFT force field at the B3YLP/DGZVP level of theory for optimization and frequency. ECD calculations in MeOH solution were carried out by employing a TD-SCF force field at the B3LYP/6-31G(d) theory level, with the default solvent model. The calculated excitation energy (nm) and rotatory strength (R) in dipole velocity (Rvel) form were simulated into an ECD curve using Equation (1), as implemented in the SpecDis software (Version 1.71), where E_0k_ and R_0k_ are the transition energy and rotatory strength of kth electronic transition, respectively, and σ is the exponential half width [43,44].

(1) $$\Delta \varepsilon =\frac{1}{2.296\times 10^{-39}}\times \frac{1}{\sigma \sqrt{\pi }}\sum_{k}E_{0k}R_{0k}e\left[-{\left\{\frac{E-E_{0k}}{\sigma }\right\}}^{2}\right]$$

All calculations were performed on the HP Cluster Platform 3000SL “Miztli”, a parallel supercomputer with a Linux operating system, containing 25,312 cores and a total of 45,000 GB of RAM.

### 3.6. Cytotoxicity Assay

Compounds **5**, **6** and **8** were evaluated in vitro against human lung adenocarcinoma (SKLU-1), breast cancer (MCF-7), human prostate cancer (PC-3), human colon cancer (HCT-15), human chronic myelogenous leukemia (K562), human glioblastoma (U251) and healthy monkey kidney (COS-7) cell lines, which were supplied by the National Cancer Institute (USA) and American Type Culture Collection (ATCC). The human tumor cytotoxicity was determined using the protein-binding dye sulforhodamine B (SRB) in a microculture assay to measure cell growth, and the assay was performed as reported in [45].

### 3.7. Phytotoxic Assay

Compounds **5**–**7**, **9** and **10** were tested for their inhibitory effects on the elongation of the roots of the seedlings of three dicotyledonous species, *Trifolium pratense* (Fabaceae) (red clover, peavine clover and cow grass), *Medicago sativa* (Fabaceae) (California clover and buffalo grass) and *Amaranthus hypochondriacus* (Amaranthaceae) (amaranth), and one monocotyledonous plant, *Panicum miliaceum* (Poaceae) (red millet). Seeds were obtained from Casa Cobo, S.A. de C.V. (Central de Abastos, Mexico City, Mexico). The assay was performed as reported in Refs. [46,47]. Briefly, a Petri dish bioassay was performed to evaluate the phytotoxic effects of different treatments on seedling growth. The compounds were evaluated at 100 μg/mL by dilution in agar (1%). The compounds were dissolved in MeOH, not exceeding 0.5%, and added to ~40 °C sterile agar in 5 cm Petri dishes before its solidification. Rival [glyphosate, N-(phosphonomethyl)glycine] (Monsanto, Sao Paulo, Brazil) at 200 μg/mL was used as a positive control; agar (1% with 0.5% MeOH) and pure agar (1%) were used as negative controls. Thirty seeds of every plant species were sown onto the agar in four replicates in a completely randomized design. The agar plates were placed in a germination chamber at 27 °C under complete darkness. After treatment, germination of the seed and root growth were measured for *A. hypochondriacus* (24 h) and *T. pratense*, *M. sativa* and *P. miliaceum* (48 h). Experimental results were analyzed by analysis of variance (ANOVA) and Tukey’s statistical tests utilizing GraphPad Prism version 6.01 statistical computer software (GraphPad Software, Inc., La Jolla, CA, USA). Data are represented as the mean ± standard deviation (SD). A *p*-value of ≤0.05 (*) was employed to indicate statistical significance.

## 4. Conclusions

From the dichloromethane extract of *S. carranzae*, three unpublished icetexane diterpenoids (**1**–**3**), named salvicarranzanolide (**1**), 19-deoxo-salvicarranzanolide (**2**) and 19-deoxo-20-deoxi-salvicarranzanolide (**3**), were isolated together with six known icetexane types (**4**–**9**), two abietanes (**10**–**11**) and α-tocopherol. Compounds **5** and **6** showed to have significant activity with an IC_50_ comparable with that of adriamycin, but a low selectivity index (SI) on U251, K562, HCT-15 and SKLU-1 human cancer cell lines. While none of the tested compounds (**5**–**7** and **9**, **10**) showed activity in the inhibition of germination, compound **5** showed moderate inhibition on *A. hypochondriacus* root elongation, and the icetexane **10** showed weak inhibition of *A. hypochondriacus* and *P*. *miliaceum* root elongation. 

The chemical profile found for *S. carranzae* reinforces its chemical relationship with species of the section *Erythrostachys*. It is necessary to carry out more chemical and phylogenetic studies on *S. carranzae* and its related species to establish its most adequate taxonomic classification. 

## Figures and Tables

**Table 1:** NMR data (^1^H 700 MHz, CDCl_3_) of **1**–**3**.

1				2			3 *		
Position	δ_C_	Type	δ_H_ (*J* in Hz)	δ_C_	Type	δ_H_ (*J* in Hz)	δ_C_	Type	δ_H_ (*J* in Hz)
1a	25.0	CH_2_	2.54, dd (13.5, 6.1)	28.6	CH_2_	2.28, dd (13.6, 6.2)	37.6	CH_2_	1.81, dd (13.4, 6.4)
1b			1.64, m		CH_2_	1.52, dd (13.6, 6.0)			1.60, td (12.9, 6.1)
2a	19.1	CH_2_	1.97, dt (13.5, 6.1)	19.6	CH_2_	1.60, m	21.1	CH_2_	1.90, dh (19.6, 6.3)
2b			1.75, m			1.49, m			1.66, m
3a	36.2	CH_2_	1.81, m	39.7	CH_2_	1.80, m	40.3	CH_2_	1.53, dd (13.3, 6.4)
3b			1.65, m			1.84, m			1.45, tdd (13.3, 5.8, 2.2)
4	47.6	C		44.1	C		44.9	C	
5	50.1	CH	2.11, dd (12.4, 1.5)	51.5	CH	1.77, dd (10.8, 3.6)	53.4	CH	1.65, m
6a	39.9	CH_2_	2.72, dd (17.5, 12.4)	40.4	CH_2_	2.78, m	41.9	CH_2_	2.95, dd (16.8, 12.4)
6b			2.80, dd (17.5, 1.5)						2.75, d (16.8)
7a	202.7	C		204.6	C		208.1	C	
7b							126.3	C	
8	109.8	C		110.1	C		113.6	C	
9	114.1	C		117.0	C		88.0	C	
10	87.5	C		88.8	C		136.4	C	
11	136.9	C		136.3	C		154.9	C	
12	151.9	C		151.4	C		120.3	C	
13	121.2	C		120.4	C		160.5	C	
14	158.8	C		158.6	C		25.7	CH	3.51, hept (7.1)
15	24.5	CH	3.57, hept (7.1)	24.5	CH	3.55, hept (7.1)	20.5	CH_3_	1.31, d (7.1)
16	20.1	CH_3_	1.34, d (7.1)	20.17	CH_3_	1.32, d (7.1)	20.6	CH_3_	1.30, d (7.1)
17	19.8	CH_3_	1.35, d (7.1)	20.22	CH_3_	1.33, d (7.1)	19.2	CH_3_	1.02, s
18	16.9	CH_3_	1.18, s	28.5	CH_3_	1.00, s	78.2	CH_2_	3.86, d (7.7)
19a	178.8	C		77.9	CH_2_	3.90, d (7.9)			3.72, dd (7.7, 2.2)
19b						3.72, dd (7.9, 2.1)	35.9	CH_2_	3.44, d (13.6)
20a	76.4	CH	5.44, s	78.5	CH	5.32, s			2.84, d (13.6)
11-OH			9.30, s			9.20, brs			
12-OH			6.69, s			6.57, brs			
14-OH			12.76, s			12.92, s			

* CD_3_OD was used as a solvent.

**Table 2:** IC_50_ (μM) values of antiproliferative activity for compounds **5** and **6**.

Compound		IC_50_ (μM) (SI)			
	U251	K562	HCT-15	SKLU-1	COS-7
**5**	0.43 ± 0.01 (2.8)	0.45 ± 0.01 (2.7)	0.84 ± 0.07 (1.4)	0.73 ± 0.06 (1.7)	1.21 ± 0.1
**6**	1.34 ± 0.04 (0.7)	1.29 ± 0.06 (0.7)	1.03 ± 0.10 (0.9)	0.95 ± 0.09 (1.0)	0.91 ± 0.05
Adriamycin	0.08 ± 0.003 (3.1)	0.20 ± 0.02 (12.5)	0.16 ± 0.01 (1.6)	0.20 ± 0.02 (1.3)	0.25 ± 0.009

Results represent the mean ± SD of at least three different experiments; U251 = human glioblastoma; K562 = human chronic myelogenous leukemia; HCT-15 = human colon cancer; SKLU-1 = human lung adenocarcinoma; COS-7 = normal monkey kidney; SI = selectivity index calculated as the quotient of the IC_50_ of COS-7/IC_50_ of cancer cell lines.

## Data Availability

The data presented in this study are available in this article.

## Supplementary Materials

The following supporting information can be downloaded at: https://www.mdpi.com/article/10.3390/molecules29061226/s1, Figures S1–S28: 1D, 2D NMR and HR-MS spectra of compounds **1**–**3** and **5**. Figure S29: Isomerization from isoicetexone to icetexone at room temperature; Table S1: Primary screening of compounds 5, 6 and 8 on antiproliferative activity; Table S2: Inhibitory growth activity of compounds **5**–**7**, **9** and **10** on the root elongation and seed germination of *Amaranthus hypochondriacus*, *Trifolium pratense*, *Medicago sativa* and *Panicum miliaceum*.

## References

[1] Drew B.T. (2020). Evolution, Pollination Biology, and Species Richness of *Salvia* (Lamiaceae). Int. J. Plant Sci..

[2] Bentham G., Bentham G., Hooker J.D. (1876). Labiatae. Genera Plantarum.

[3] Esquivel B., Sánchez A.A., Aranda E., Shahidi F., Ho C. (2000). Natural Products of Agrochemical Interest from Mexican Labiatae. Phytochemicals and Phytopharmaceuticals.

[4] Harley R.M., Heywood C., Harley R.M., Reynolds T. (1992). Chromosome Numbers in Tropical American Labiatae. Advances in Labiatae Science.

[5] Kriebel R., Drew B.T., Drummond C.P., González-Gallegos J.G., Celep F., Mahdjoub M.M., Rose J.P., Xiang C.L., Hu G.X., Walker J.B. (2019). Tracking Temporal Shifts in Area, Biomes, and Pollinators in the Radiation of *Salvia* (Sages) across Continents: Leveraging Anchored Hybrid Enrichment and Targeted Sequence Data. Am. J. Bot..

[6] Drew B.T., González-Gallegos J.G., Xiang C.L., Kriebel R., Drummond C.P., Walker J.B., Sytsma K.J. (2017). *Salvia* United: The Greatest Good for the Greatest Number. Taxon.

[7] Will M., Claßen-Bockhoff R. (2017). Time to Split Salvia s.l. (Lamiaceae)—New Insights from Old World *Salvia* Phylogeny. Mol. Phylogenet. Evol..

[8] Hu G.X., Takano A., Drew B.T., Liu E.D., Soltis D.E., Soltis P.S., Peng H., Xiang C.L. (2018). Phylogeny and Staminal Evolution of *Salvia* (Lamiaceae, Nepetoideae) in East Asia. Ann. Bot..

[9] Villaseñor J.L. (2016). Catálogo de Las Plantas Vasculares Nativas de México. Rev. Mex. Biodivers..

[10] Martínez-Gordillo M., Bedolla-García B., Cornejo-Tenorio G., Fragoso-Martínez I., García-Peña M.D.R., González-Gallegos J.G., Lara-Cabrera S.I., Zamudio S. (2017). Lamiaceae de México. Bot. Sci..

[11] Ramamoorthy T.P., Calderón de Rzedowski G., Rzedowski J. (2005). Salvia L. Flora fanerogámica del Valle de México.

[12] González-Gallegos J.G., Castro-Castro A., Quintero-Fuentes V., Mendoza-López M.E., De Castro-Arce E. (2016). Revisión Taxonómica de Lamiceae Del Occidente de México. Ibugana.

[13] González-Gallegos J.G., Bedolla-García B.Y., Cornejo-Tenorio G., Fernández-Alonso J.L., Fragoso-Martínez I., García-Peña M.D.R., Harley R.M., Klitgaard B., Martínez-Gordillo M.J., Wood J.R.I. (2020). Richness and Distribution of *Salvia* Subg. *Calosphace* (Lamiaceae). Int. J. Plant Sci..

[14] Bedolla-García B.Y., Zamudio S., Castillo-Gómez H.A. (2020). *Salvia huastecana* (Lamiaceae), a New Species from San Luis Potosí, Mexico. Phytotaxa.

[15] González-Gallegos J.G., Castro-Castro A., Ávila-González H. (2020). *Salvia rhizomatosa* (Lamiaceae) a New Species from Sierra Madre Occidental in Durango, Mexico, with a Synopsis of *Salvia* Sect. Brandegeia. Phytotaxa.

[16] Fragoso-Martínez I., Martínez-Gordillo M., Salas S. (2021). *Salvia fimbriaticalyx*, a New Species of *Salvia* (Lamiaceae) from Oaxaca, Mexico. Phytotaxa.

[17] González-Gallegos J.G., Pío-León J.F., Castro-Castro A. (2021). *Salvia beltraniorum* (Lamiaceae), a New Species in Savannoid Vegetation from Cosalá, Sinaloa, Mexico. Phytotaxa.

[18] González-Gallegos J.G., Bedolla-García B.Y., Uría R. (2021). *Salvia gomezpompae* (Lamiaceae), a New Species from Veracruz, Mexico. Bot. Sci..

[19] Bedolla-García B.Y., Zamudio S. (2015). Four New Species of *Salvia* (Lamiaceae) from Central Mexico. Phytotaxa.

[20] Epling C. (1939). A Revision of *Salvia* Subgenus *Calosphace*. Feddes Repert. Specierum Nov. Regni Veg..

[21] Esquivel B., Cardenas J., Toscano A., Soriano-García M., Rodríguez-Hahn L. (1985). Structure of Salvigenolide, a Novel Diterpenoid with a Rearranged Neo-Clerodane Skeleton from *Salvia fulgens*. Tetrahedron.

[22] Esquivel B., Cardenas J., Rodriguez-Hahn L., Ramamoorthy T.P. (1987). The Diterpenoid Constituents of *Salvia fulgens* and *Salvia microphylla*. J. Nat. Prod..

[23] Narukawa Y., Fukui M., Hatano K., Takeda T. (2006). Four New Diterpenoids from *Salvia fulgens* Cav. J. Nat. Med..

[24] Esquivel B., Cardenas J., Ramamoorthy T.P., Rodriguez-Hahn L. (1986). Clerodane Diterpenoids of *Salvia lineata*. Phytochemistry.

[25] Esquivel B., Martínez N.D.S., Cárdenas J., Ramamoorthy T.P., Rodríguez-Hahn L. (1989). The Pimarane-Type Diterpenoids of *Salvia microphylla* Var. Neurepia. Planta Med..

[26] Bautista E., Toscano R.A., Ortega A. (2013). Microphyllandiolide, a New Diterpene with an Unprecedented Skeleton from *Salvia microphylla*. Org. Lett..

[27] Bautista E., Toscano R.A., Ortega A. (2014). 5,10- Seco—Neo -Clerodanes and Neo -Clerodanes from *Salvia microphylla*. J. Nat. Prod..

[28] Esquivel B., Bustos-Brito C., Sánchez-Castellanos M., Nieto-Camacho A., Ramírez-Apan T., Joseph-Nathan P., Quijano L. (2017). Structure, Absolute Configuration, & Antiproliferative Activity of Abietane & Icetexane Diterpenoids from *Salvia ballotiflora*. Molecules.

[29] Cezarotto C.S., Dorneles A., Baldissera F.G., da Silva M.B., Markoski M.M., Júnior L.C.R., Peres A., Fazolo T., Bordignon S.A.L., Apel M.A. (2019). Leishmanicidal and Antichemotactic Activities of Icetexanes from *Salvia uliginosa* Benth. Phytomedicine.

[30] Campos-Xolalpa N., Alonso-Castro Á.J., Sánchez-Mendoza E., Zavala-Sánchez M.Á., Pérez-Gutiérrez S. (2017). Cytotoxic Activity of the Chloroform Extract and Four Diterpenes Isolated from *Salvia ballotiflora*. Rev. Bras. Farmacogn..

[31] Esquivel B., Burgueño-Tapia E., Bustos-Brito C., Pérez-Hernández N., Quijano L., Joseph-Nathan P. (2018). Absolute Configuration of the Diterpenoids Icetexone and Conacytone from *Salvia ballotaeflora*. Chirality.

[32] Brandt C.W., Neubauer L.G. (1939). 221. Miro Resin. Part I. Ferruginol. J. Chem. Soc..

[33] González M.A. (2015). Aromatic Abietane Diterpenoids: Their Biological Activity and Synthesis. Nat. Prod. Rep..

[34] Gómez-Rivera A., González-Cortazar M., Herrera-Ruíz M., Zamilpa A., Rodríguez-López V. (2018). Sessein and Isosessein with Anti-Inflammatory, Antibacterial and Antioxidant Activity Isolated from *Salvia sessei* Benth. J. Ethnopharmacol..

[35] Jimenez E.M., Portugal M.E., Lira-Rocha A., Soriano-Garcia M., Toscano R.A. (1988). A New Royleanone-Type Diterpene from *Salvia sessei*. J. Nat. Prod..

[36] Hernández M., Esquivel B., Cárdenas J., Rodríguez-Hahn L., Ramamoorthy T.P. (1987). Diterpenoid Abietane Quinones Isolated from *Salvia regla*. Phytochemistry.

[37] Ortega A., Cárdenas J., Gage D.A., Maldonado E. (1995). Abietane and Clerodane Diterpenes from *Salvia regla*. Phytochemistry.

[38] Esquivel B., Calderon J.S., Flores E., Chavez C., Juarez M. (1997). Abietane and Icetexane Diterpenoids from *Salvia pubescens*. Nat. Prod. Lett..

[39] Fragoso-Martínez I., Martínez-Gordillo M., Salazar G.A., Sazatornil F., Jenks A.A., García-Peña M.D.R., Barrera-Aveleida G., Benitez-Vieyra S., Magallón S., Cornejo-Tenorio G. (2018). Phylogeny of the Neotropical Sages (*Salvia* Subg. *Calosphace*; Lamiaceae) and Insights into Pollinator and Area Shifts. Plant Syst. Evol..

[40] Jenks A.A., Walker J.B., Kim S.C. (2013). Phylogeny of New World *Salvia* Subgenus *Calosphace* (Lamiaceae) Based on CpDNA (PsbA-TrnH) and NrDNA (ITS) Sequence Data. J. Plant Res..

[41] Forzato C., Nitti P. (2022). New Diterpenes with Potential Antitumoral Activity Isolated from Plants in the Years 2017–2022. Plants.

[42] Bisio A., Fraternale D., Giacomini M., Giacomelli E., Pivetti S., Russo E., Caviglioli G., Romussi G., Ricci D., De Tommasi N. (2010). Phytotoxicity of *Salvia* spp. Exudates. Crop Prot..

[43] Gerhard B.T.A.A.H.Y.B. (2013). SpecDis: Quantifying the Comparison of Claculated and Experimental Electronic Circular Dichroism Spectra. Chirality.

[44] Bustos-Brito C., Pérez-Juanchi D., Rivera-Chávez J., Hernández-Herrera A.D., Bedolla-García B.Y., Zamudio S., Ramírez-Apan T., Quijano L., Esquivel B. (2021). Clerodane and 5 10-Seco-Clerodane-Type Diterpenoids from *Salvia involucrata*. J. Mol. Struct..

[45] Monks A., Dominic S., Skehan P., Shoemaker R., Paull K., Vistica D., Hose C., Langley J., Cronise P., Vaigro-Wolff A. (1991). Feasibility of a High-Flux Anticancer Drug Screen Using a Diverse Panel of Cultured Human Tumor Cell Lines. JNCI J. Natl. Cancer Inst..

[46] Macías-Rubalcava M.L., Ruiz-Velasco Sobrino M.E., Meléndez-González C., Hernández-Ortega S. (2014). Naphthoquinone Spiroketals and Organic Extracts from the Endophytic Fungus *Edenia gomezpompae* as Potential Herbicides. J. Agric. Food Chem..

[47] García-Méndez M.C., Macías-Ruvalcaba N.A., Lappe-Oliveras P., Hernández-Ortega S., Macías-Rubalcava M.L. (2016). Phytotoxic Potential of Secondary Metabolites and Semisynthetic Compounds from Endophytic Fungus *Xylaria feejeensis* Strain SM3e-1b Isolated from *Sapium macrocarpum*. J. Agric. Food Chem..

