# Supplementary material for: Structure, Absolute Configuration, Antiproliferative and Phytotoxic Activities of Icetexane and Abietane Diterpenoids from Salvia carranzae and Chemotaxonomic Implications
Source: Molecules. 2024 Mar 9;29(6):1226. doi: 10.3390/molecules29061226 (PMC10975418; doi:10.3390/molecules29061226)
Supplement: Supplementary file 1 [file molecules-29-01226-s001.zip › molecules-2852355-supplementary.pdf]

Article

Structure, Absolute Configuration, Antiproliferative and Phytotoxic Activities of Icetexane and Abietane Diterpenoids from *Salvia carranzae*, and Chemotaxonomic implications.

Celia Bustos Brito [1,*], Juan Pablo Torres-Medicis [1], Brenda Y. Bedolla-García [2], Sergio Zamudio [3], Teresa Ramírez-Apan [1], Martha Lydia Macías-Rubalcava [1], Leovigildo Quijano [1], and Baldomero Esquivel [1,*]

[1] Instituto de Química, Universidad Nacional Autónoma de México, Circuito Exterior, Ciudad Universitaria, Ciudad de México, 04510 México

[2] Instituto de Ecología, A. C., Centro Regional del Bajío, Apartado Postal 386, 61600 Pátzcuaro, Michoacán, México.

[3] Apartado Postal 392, 61600 Pátzcuaro, Michoacán, México.

*Correspondence: BE besquivel@iquimica.unam.mx; CBB celia.bustos@iquimica.unam.mx

Supplementary Materials

Table of Contents

**Figures S1**. ^{1}H NMR ($CDCl_3$, 700 MHz) spectrum of **1**	**4**
**Figure S2**. APT NMR ($CDCl_3$, 175 MHz) spectrum of **1**	**5**
**Figure S3**. COSY NMR ($CDCl_3$, 700 MHz) spectrum of **1**	**6**
**Figure S4**. HMBC NMR ($CDCl_3$, 700 MHz) spectrum of **1**	**7**
**Figure S5**. HSQC NMR ($CDCl_3$, 700 MHz) spectrum of **1**	**8**
**Figure S6**. NOESY NMR ($CDCl_3$, 700 MHz) spectrum of **1**	**9**
**Figure S7**. HR-DART-MS of **1**	**10**
**Figure S8**. ^{1}H NMR ($CDCl_3$, 700 MHz) spectrum of **2**	**11**
**Figure S9**. ^{13}C NMR ($CDCl_3$, 175 MHz) spectrum of **2**	**12**
**Figure S10**. COSY NMR ($CDCl_3$, 700 MHz) spectrum of **2**	**13**
**Figure S11**. HMBC NMR ($CDCl_3$, 700 MHz) spectrum of **2**	**14**
**Figure S12**. HSQC NMR ($CDCl_3$, 700 MHz) spectrum of **2**	**15**
**Figure S13**. NOESY NMR ($CDCl_3$, 700 MHz) spectrum of **2**	**16**
**Figure S14**. HR-DART-MS of **2**	**17**
**Figure S15**. ^{1}H NMR (CD_3OD, 700 MHz) spectrum of **3**	**18**
**Figure S16**. ^{13}C NMR (CD_3OD, 175 MHz) spectrum of **3**	**19**
**Figure S17**. COSY NMR (CD_3OD, 700 MHz) spectrum of **3**	**20**
**Figure S18**. HMBC NMR (CD_3OD, 700 MHz) spectrum of **3**	**21**
**Figure S19**. HSQC NMR (CD_3OD, 700 MHz) spectrum of **3**	**22**
**Figure S20**. NOESY NMR (CD_3OD, 700 MHz) spectrum of **3**	**23**
**Figure S21**. HR-DART-MS of **3**	**24**

**Figure S22**. ^{1}H NMR (CD_2Cl_2, 700 MHz) spectrum of **5**	**25**
**Figure S23**. ^{13}C NMR (CD_2Cl_2, 175 MHz) spectrum of **5**	**26**
**Figure S24**. COSY NMR (CD_2Cl_2, 700 MHz) spectrum of **5**	**27**
**Figure S25**. HMBC NMR (CD_2Cl_2, 700 MHz) spectrum of **5**	**28**
**Figure S26**. HSQC NMR (CD_2Cl_2, 700 MHz) spectrum of **5**	**29**
**Figure S27**. NOESY NMR (CD_2Cl_2, 700 MHz) spectrum of **5**	**30**
**Figure S28**. HR-DART-MS of **5**	**31**
**Figure S29**. Isomerization from isocetexone to icetexone at room temperature	**32**
**Table S1**. Primary screening of compounds **5**, **6** and **8** on antiproliferative activity at concentrations of 25 and 1.0 μM	**33**
**Table S2**. Growth inhibitory activity of compounds **5**-**7**, **9,** and **10** on the root elongation and seed germination of *Amaranthus hypochondriacus*, *Trifolium pratense*, *Medicago sativa*, and *Panicum miliaceum* at 100 μg/mL.	**34**

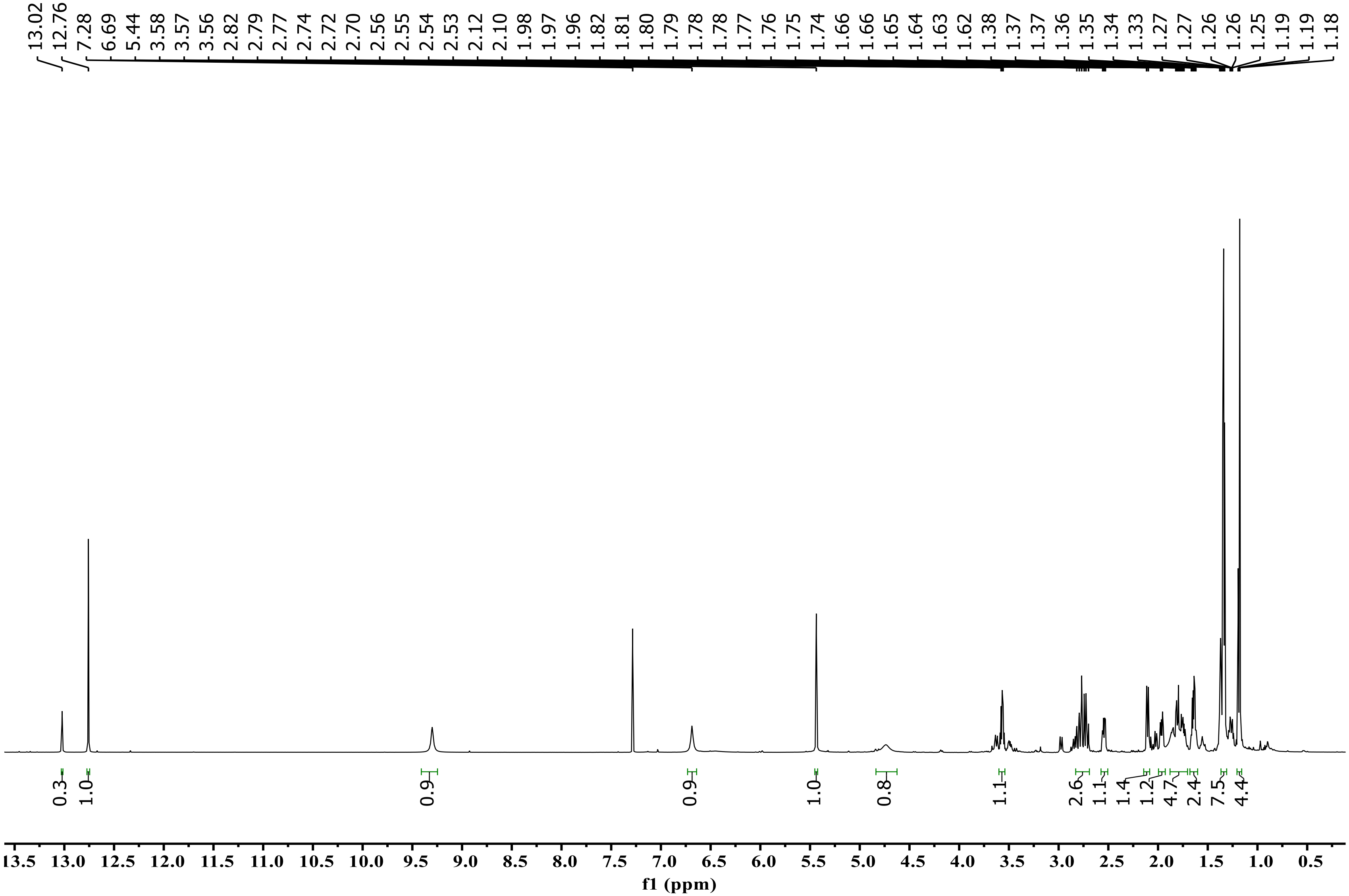

Figure S1. ^{1}H NMR ($CDCl_3$, 700 MHz) spectrum of **1**.

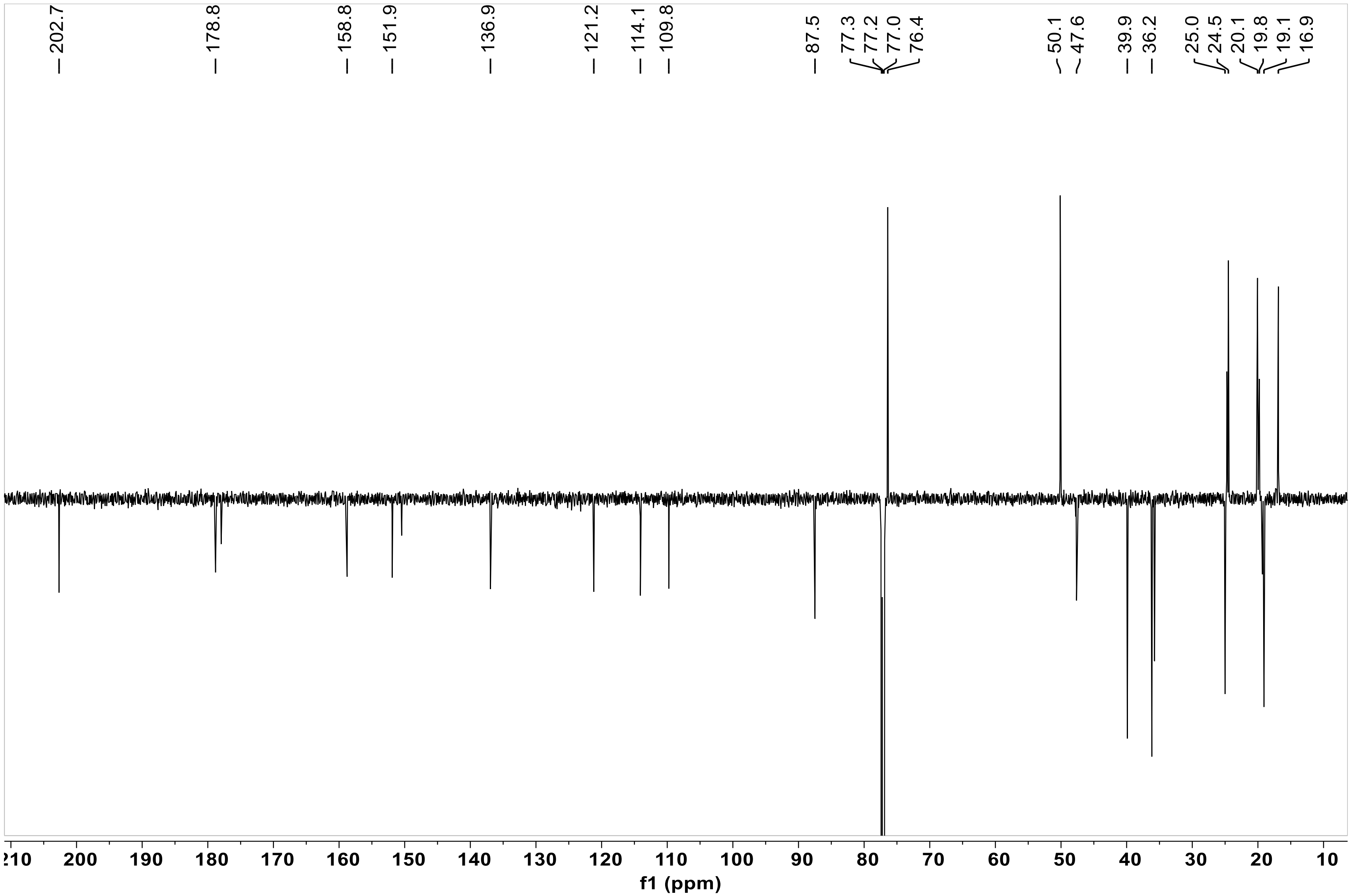

Figure S2. APT NMR ($CDCl_3$, 175 MHz) spectrum of **1**.

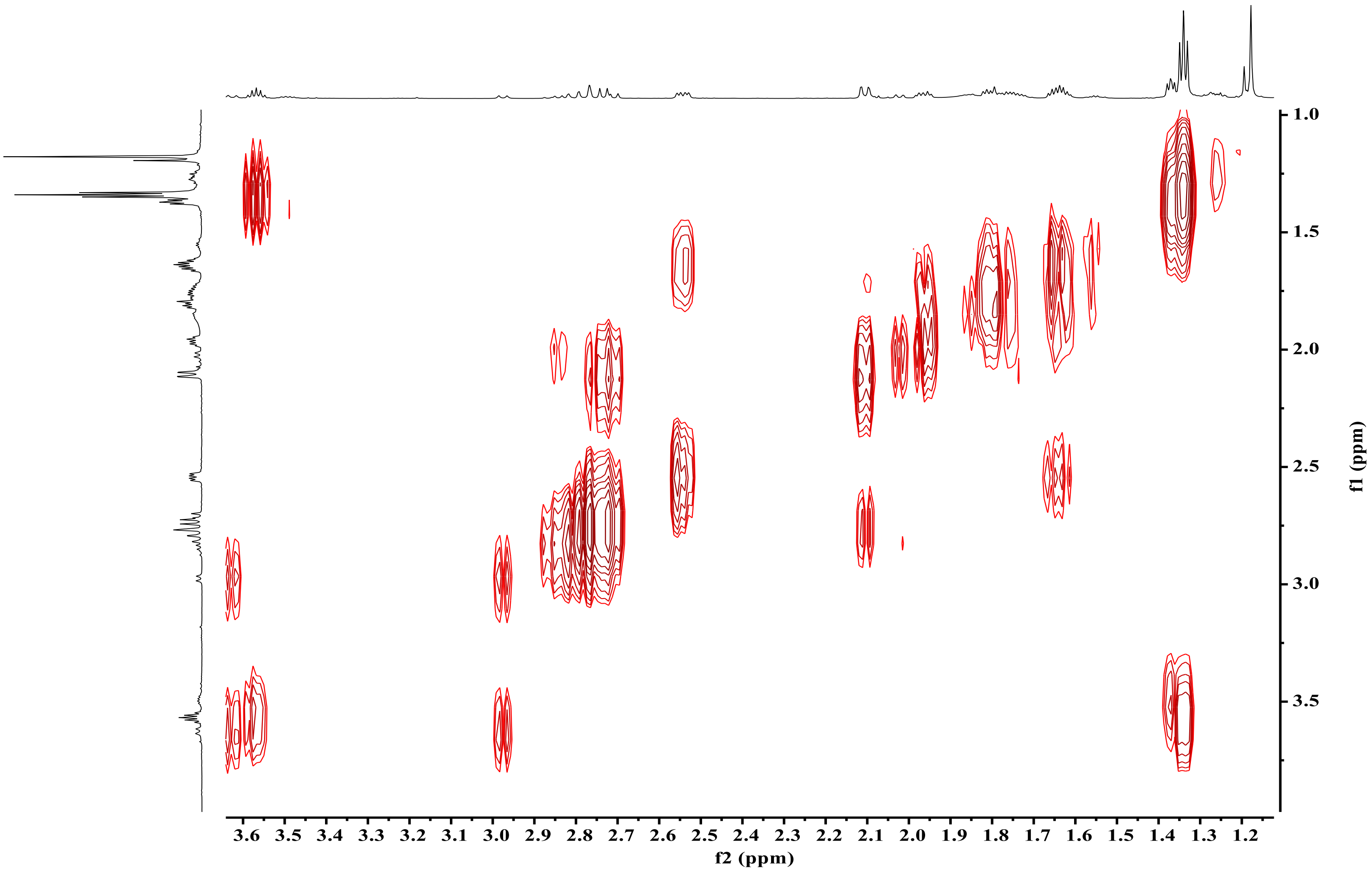

Figure S3. COSY NMR ($CDCl_3$, 700 MHz) spectrum of **1**.

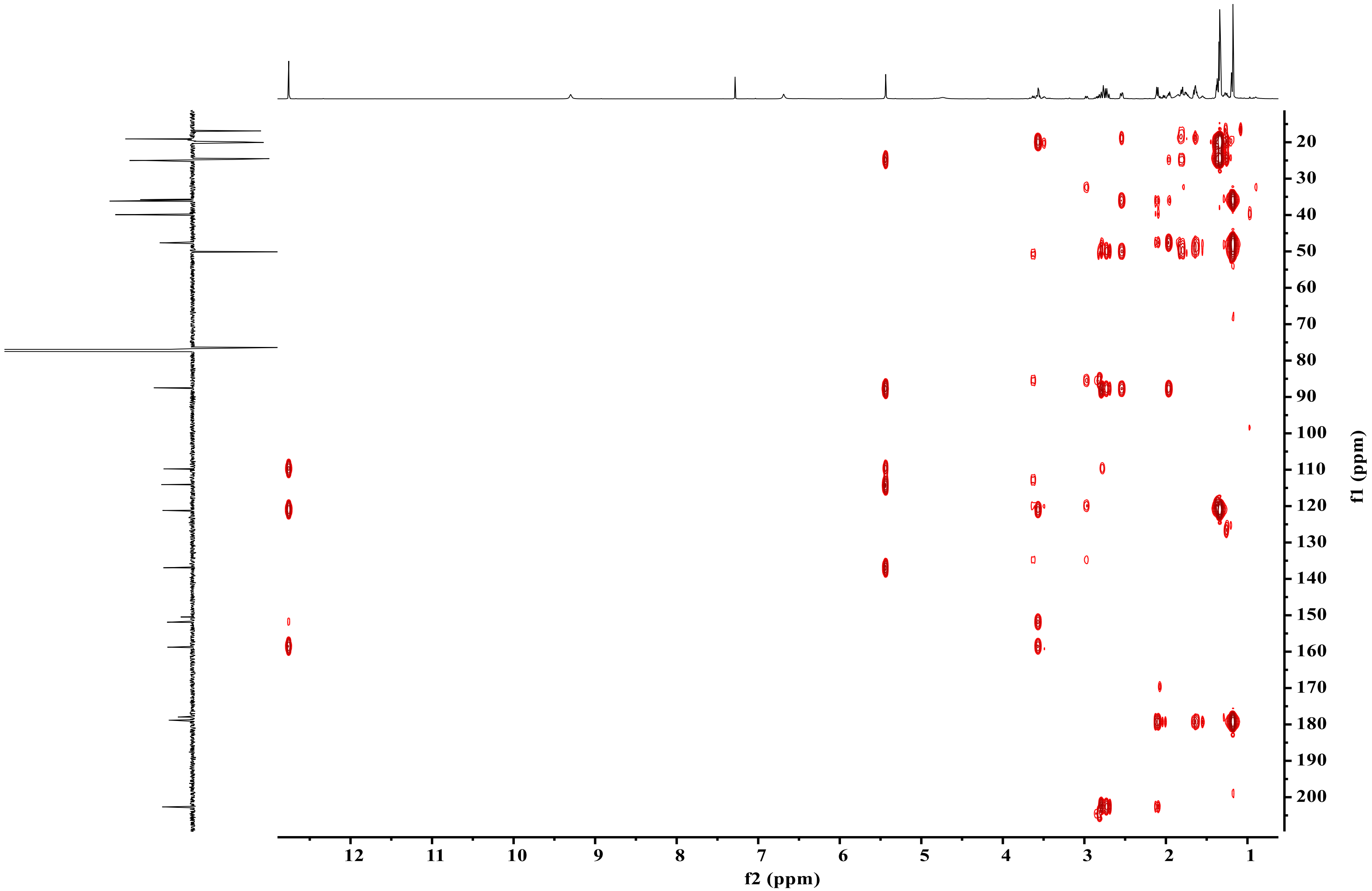

Figure S4. HMBC NMR ($CDCl_3$, 700 MHz) spectrum of **1**.

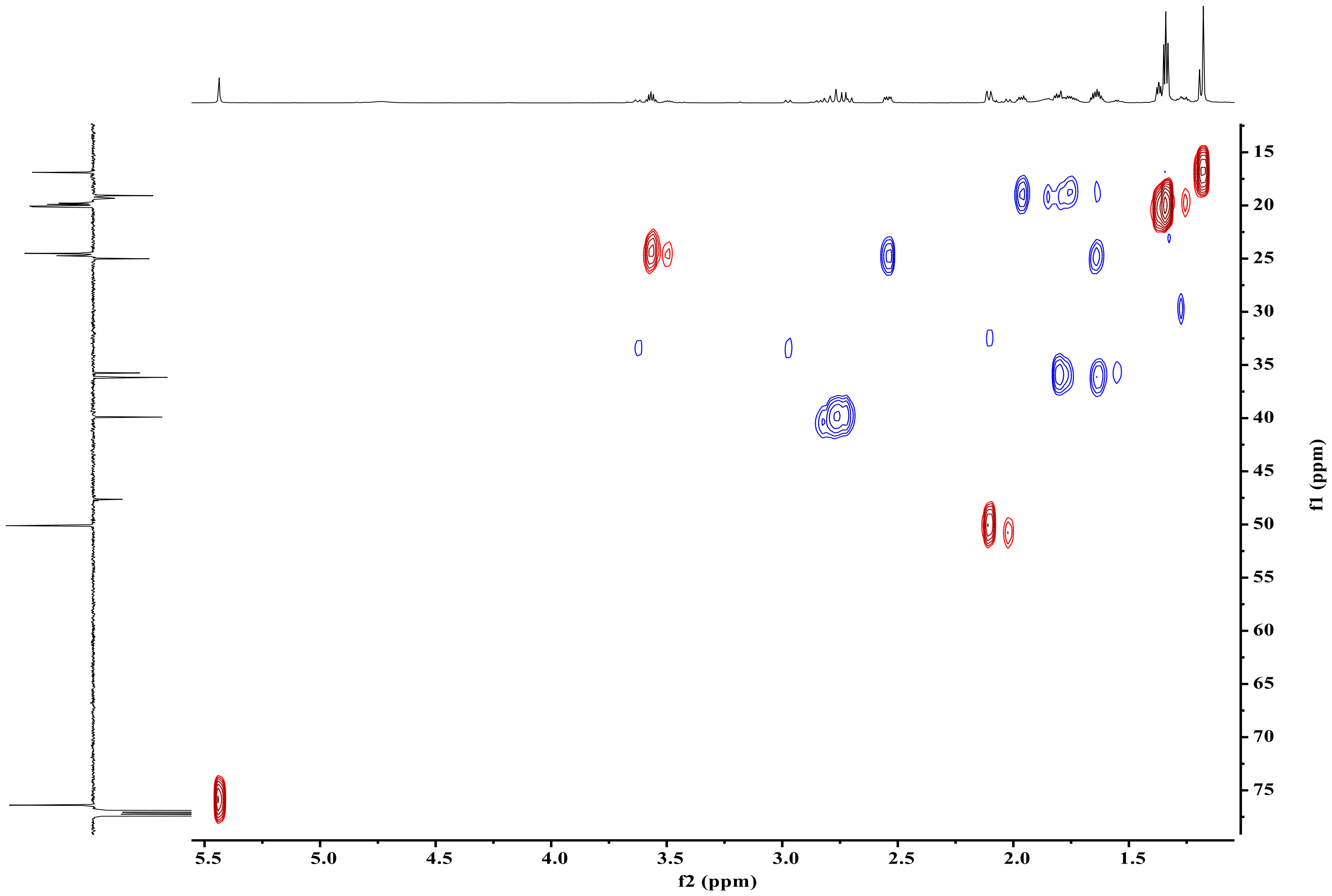

Figure S5. HSQC NMR ($CDCl_3$, 700 MHz) spectrum of **1**.

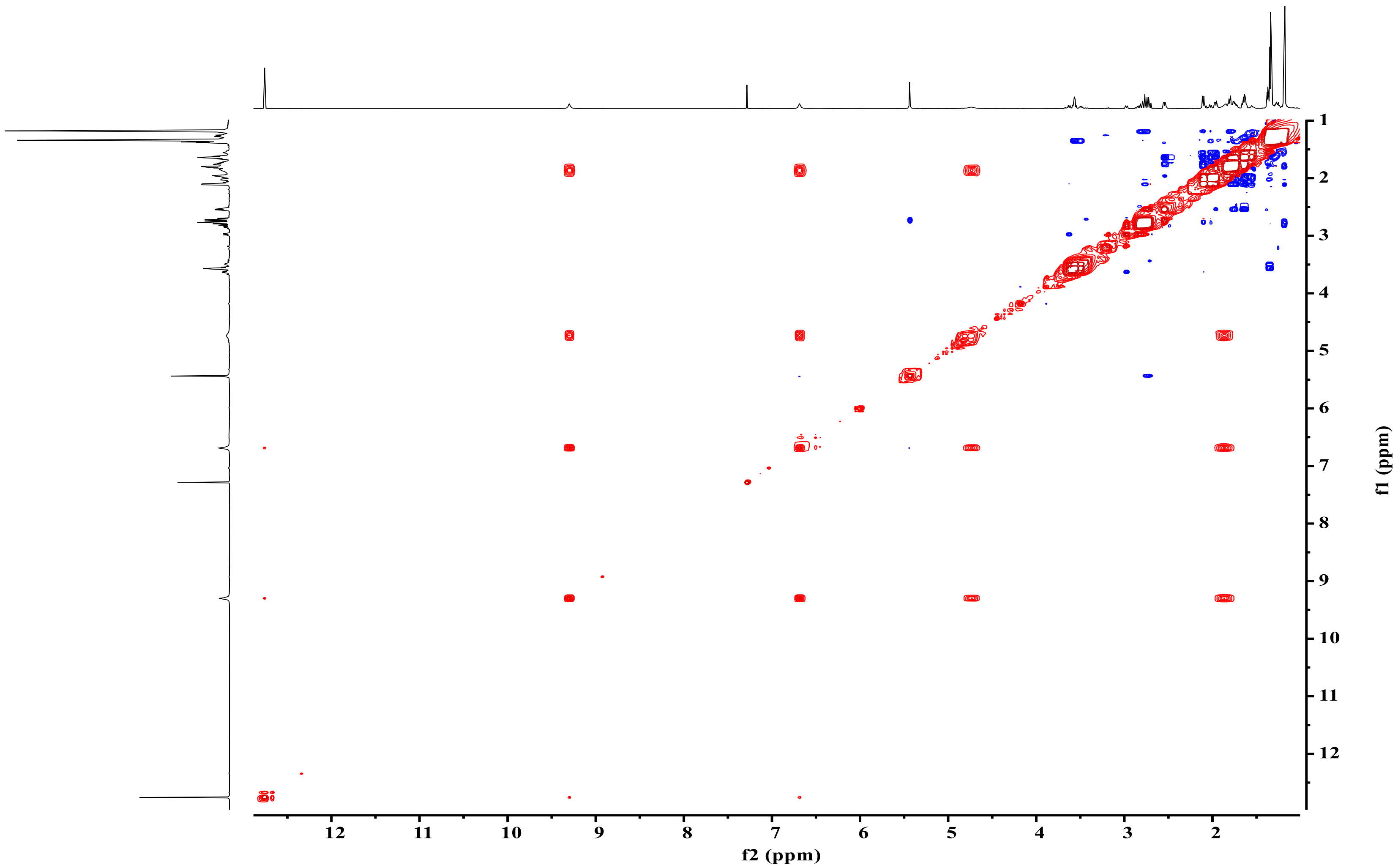

Figure S6. NOESY NMR ($CDCl_3$, 700 MHz) spectrum of **1**.

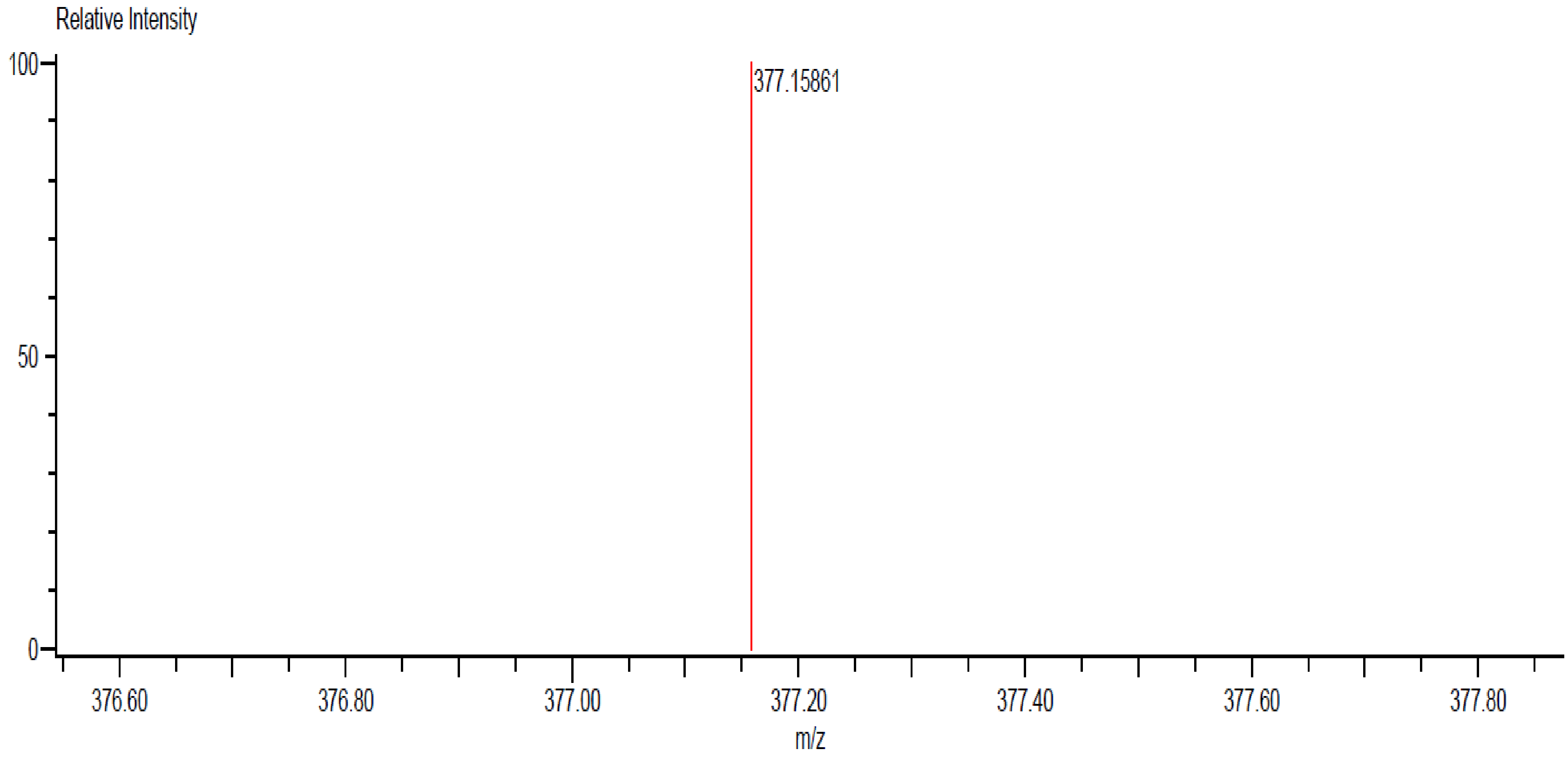

Mass	Intensity	Calc. Mass	Mass Difference (mmu)	Mass Difference (ppm)	Possible Formula	Unsaturation Number
377.15861	177099.85	377.16003	-1.42	-3.76	$^{12}C_{20}{}^{1}H_{25}{}^{16}O_{7}$	8.5

Figure S7. HR-DART-MS of **1**.

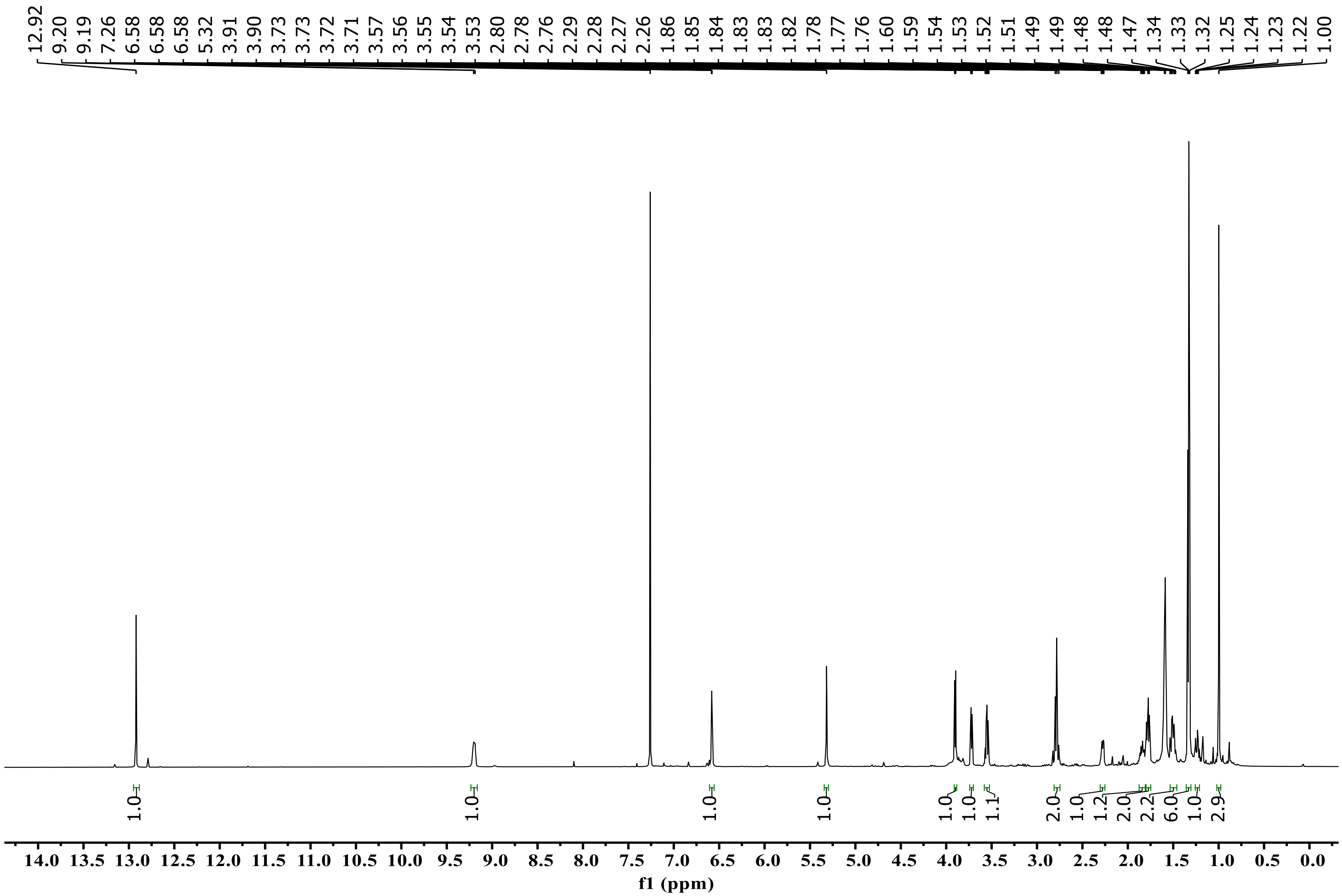

Figure S8. ^{1}H NMR ($CDCl_3$, 700 MHz) spectrum of **2**.

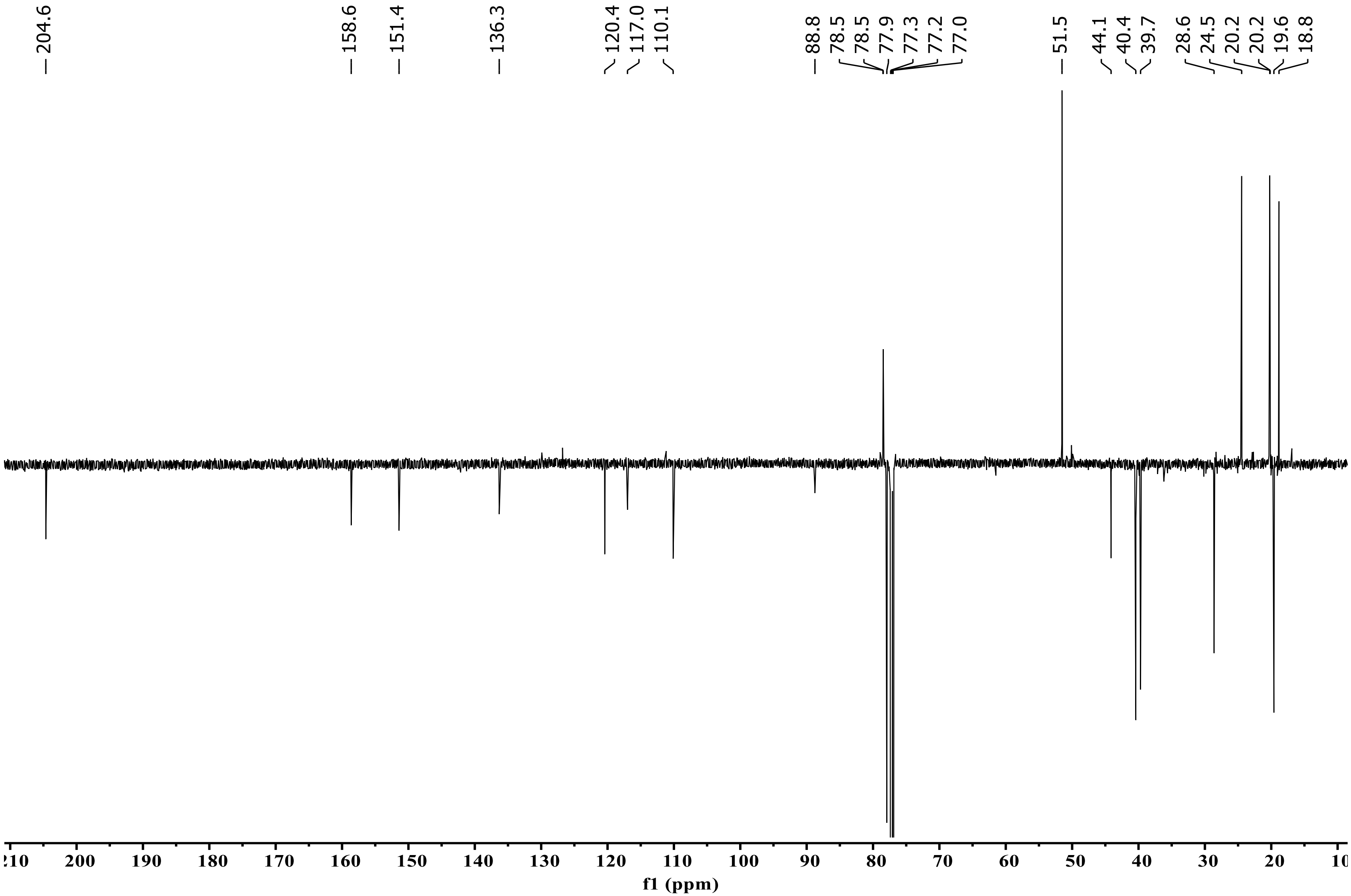

Figure S9. APT NMR ($CDCl_3$, 175 MHz) spectrum of **2**.

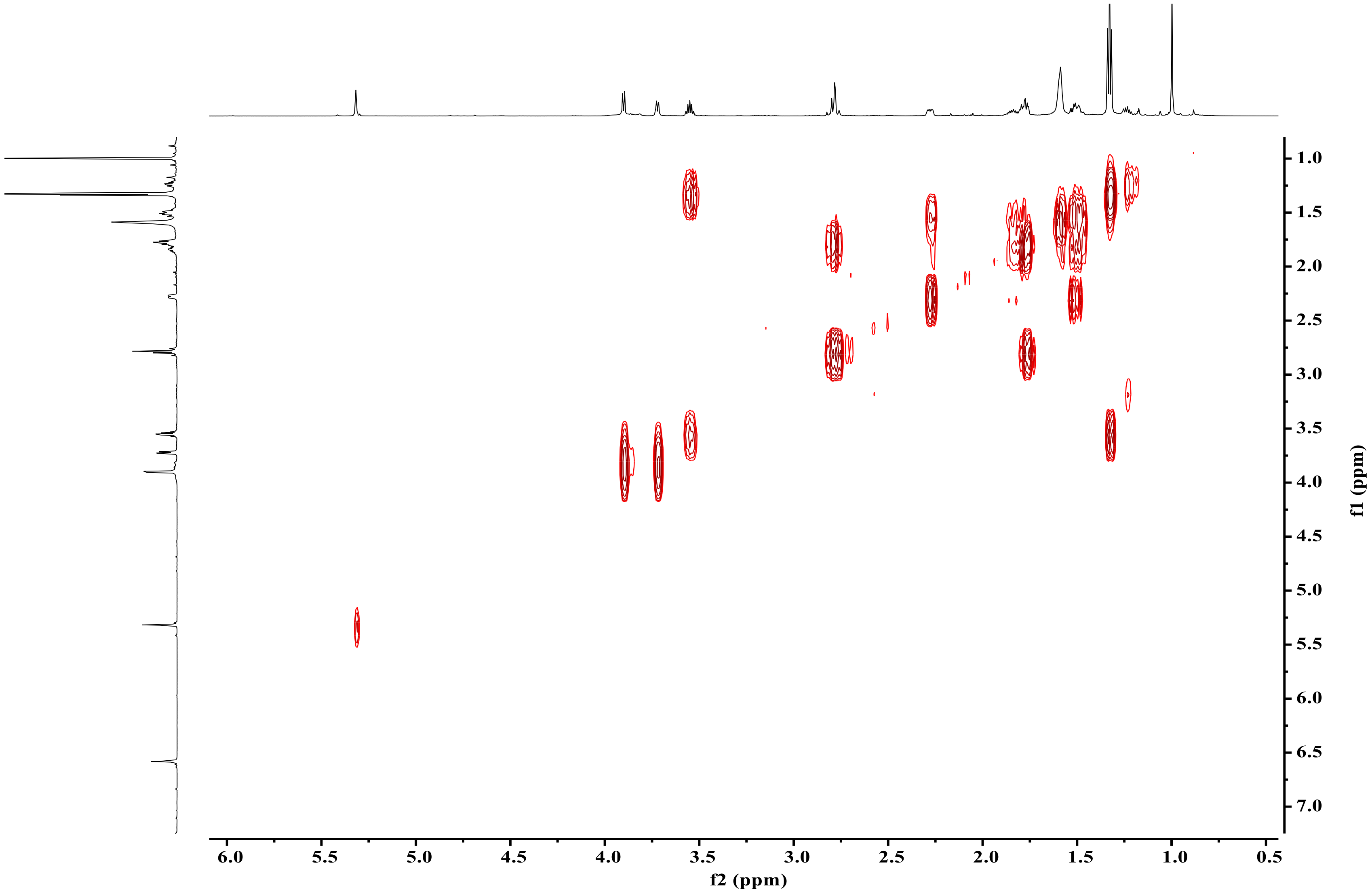

Figure S10. COSY NMR ($CDCl_3$, 700 MHz) spectrum of **2**.

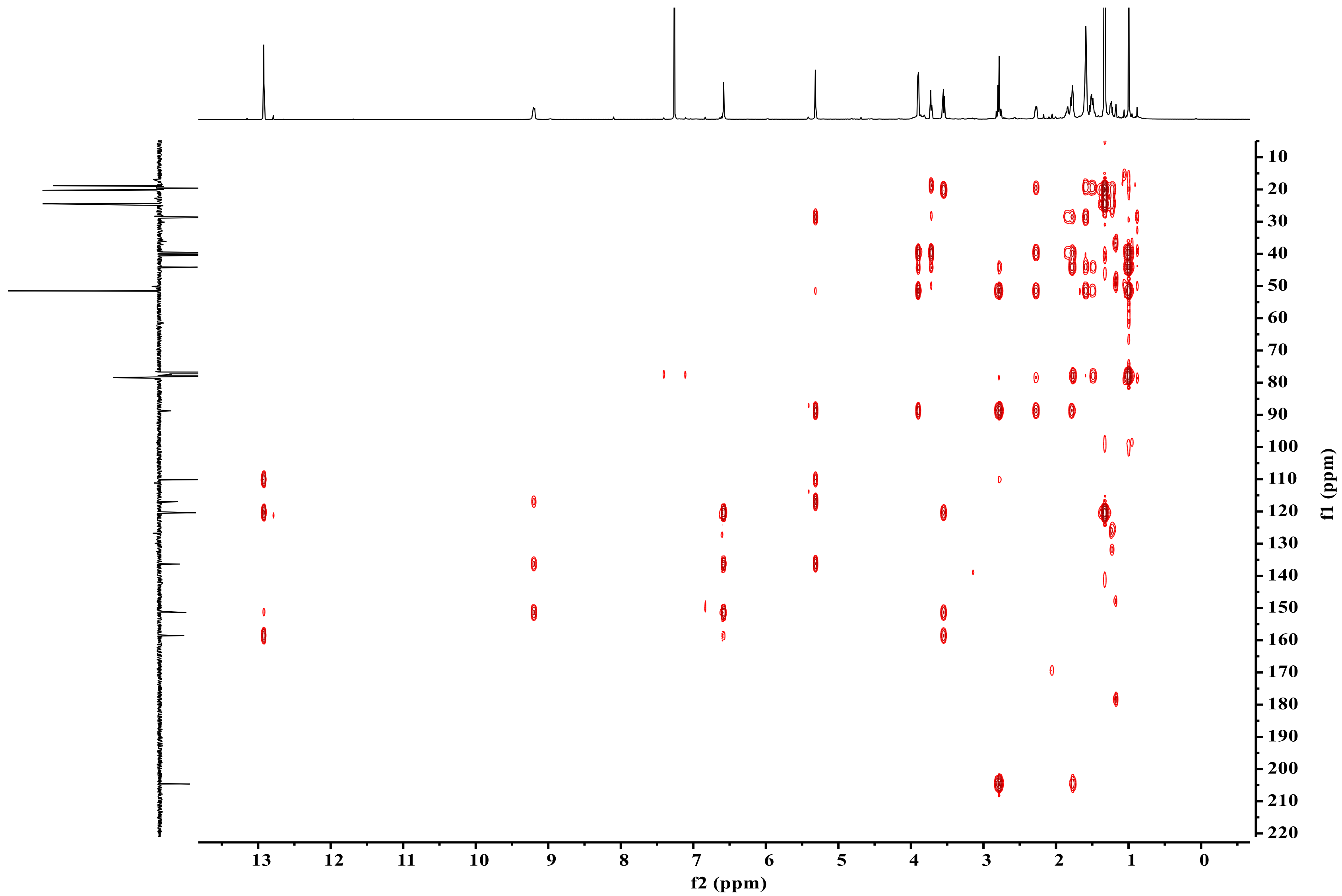

Figure S11. HMBC NMR ($CDCl_3$, 700 MHz) spectrum of **2**.

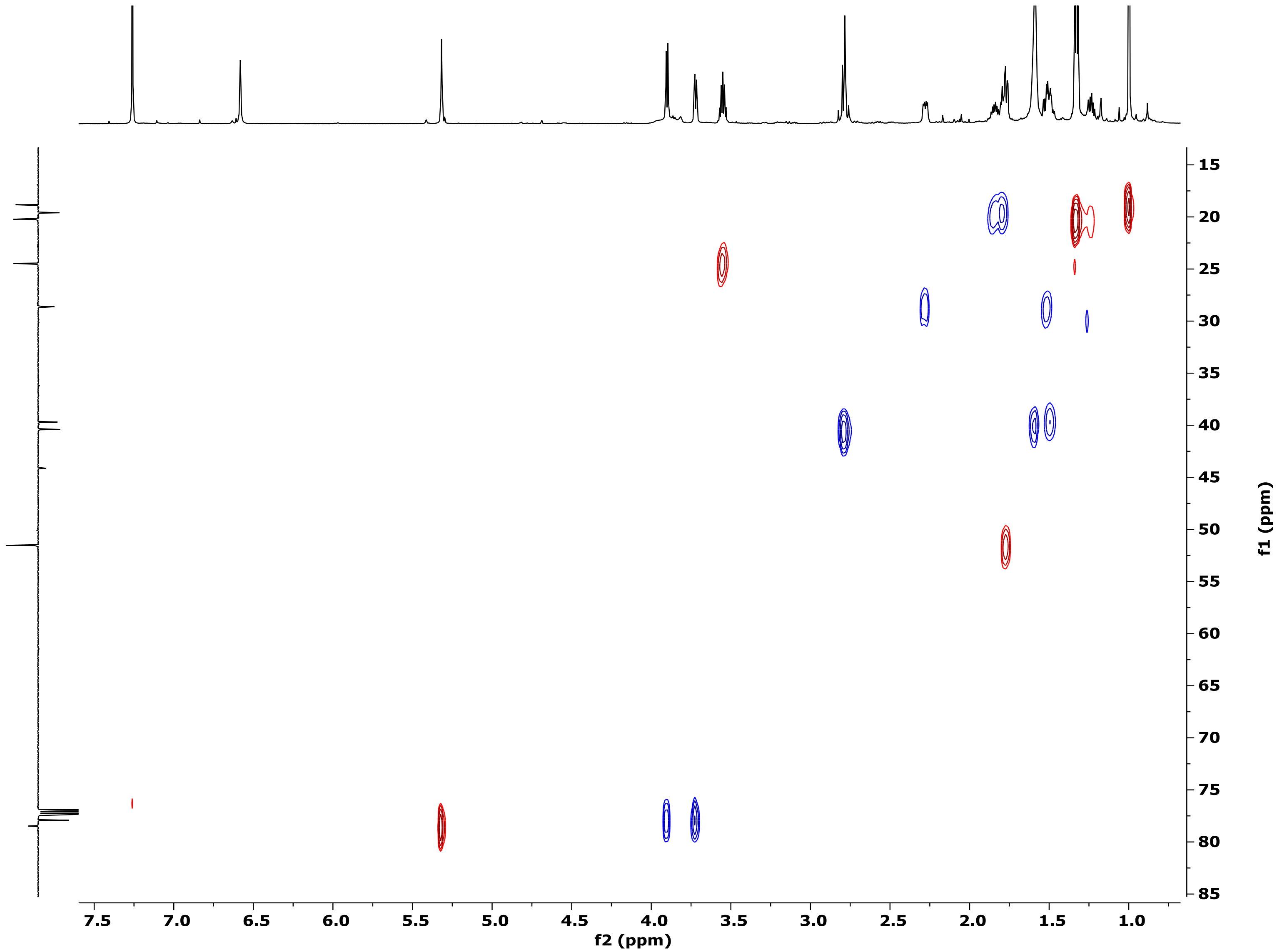

Figure S12. HSQC NMR ($CDCl_3$, 700 MHz) spectrum of **2**.

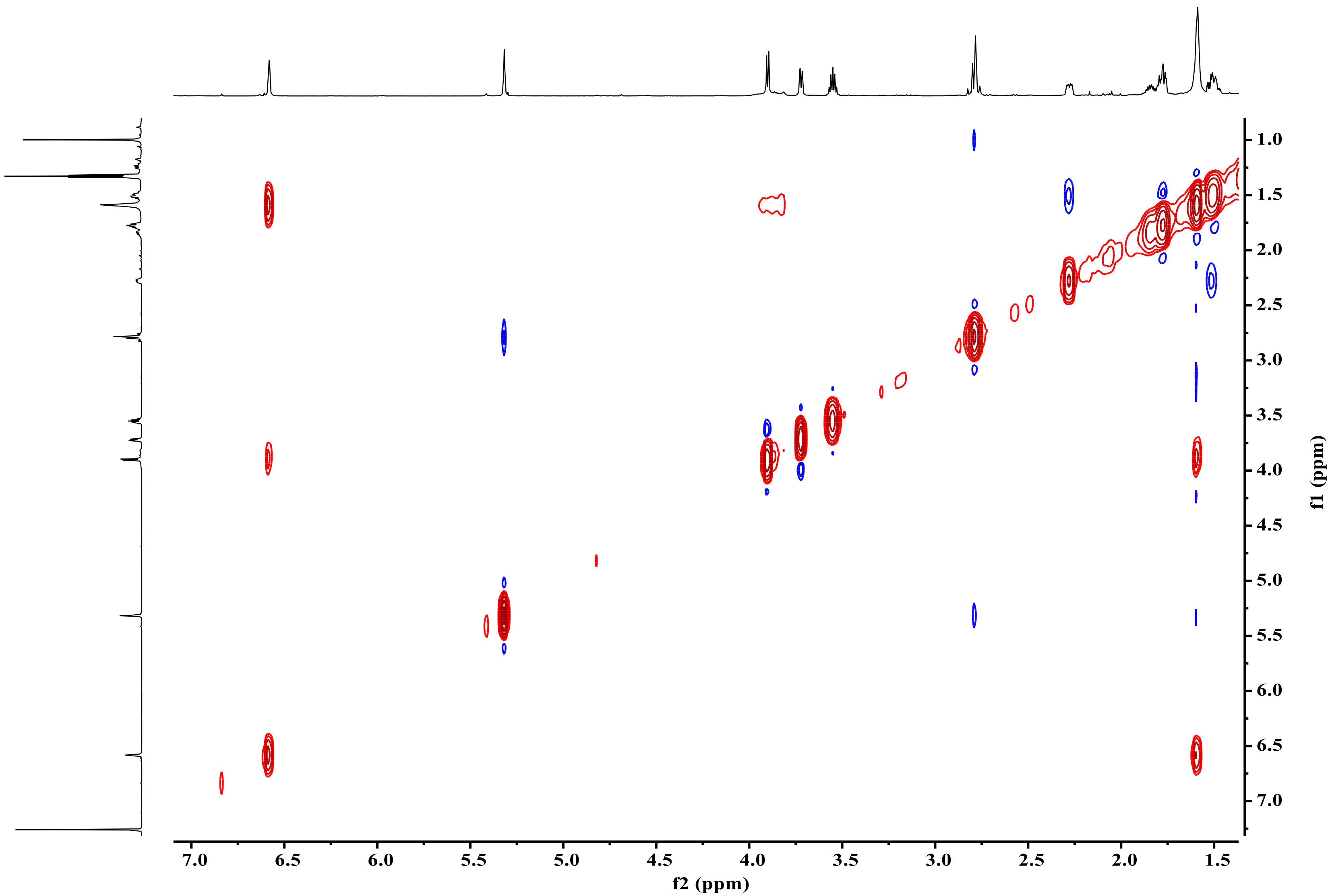

Figure S13. NOESY NMR ($CDCl_3$, 700 MHz) spectrum of **2**.

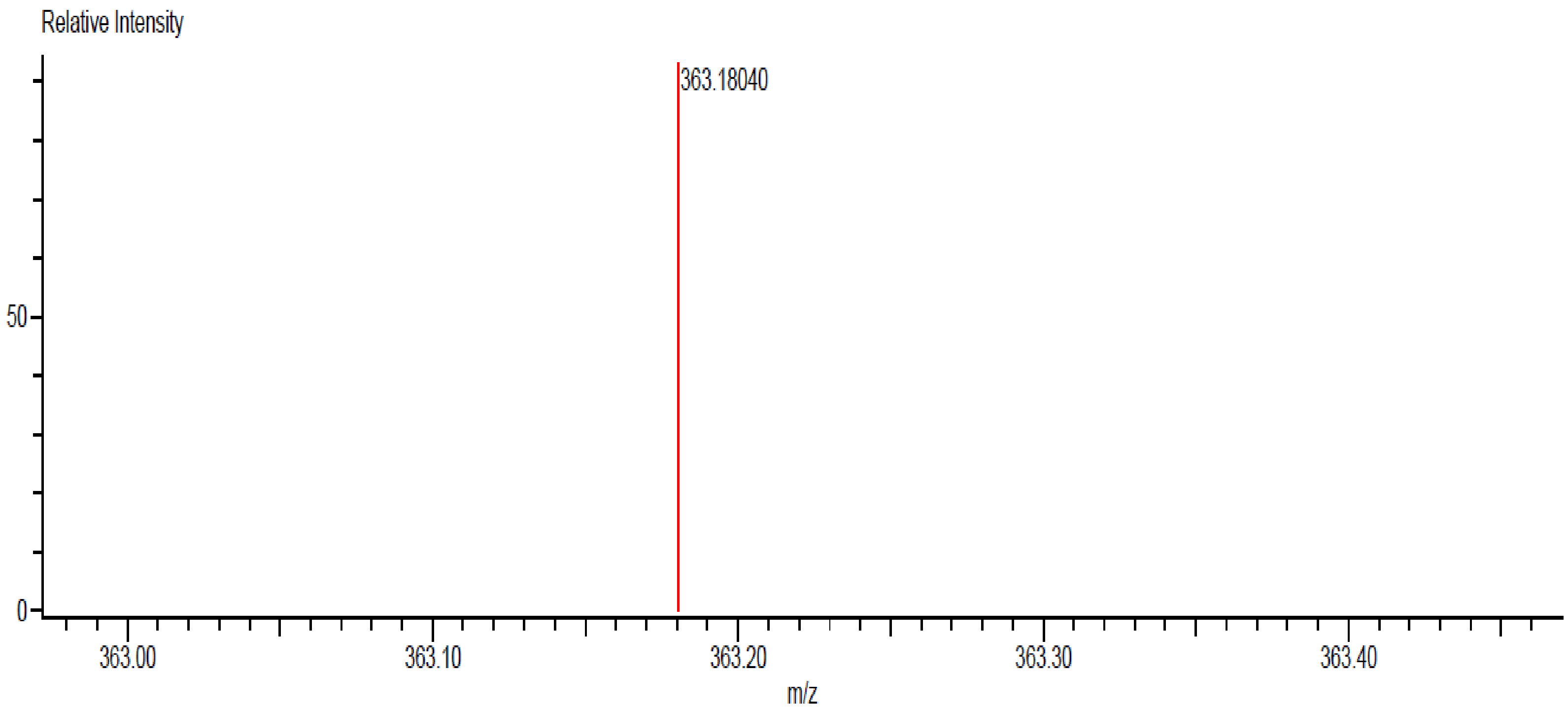

Mass	Intensity	Calc. Mass	Mass Difference (mmu)	Mass Difference (ppm)	Possible Formula	Unsaturation Number
363.18040	36767.91	363.18076	-0.37	-1.01	$^{12}C_{20}{}^{1}H_{27}{}^{16}O_{6}$	7.5

Figure S14. HR-DART-MS of **2**.

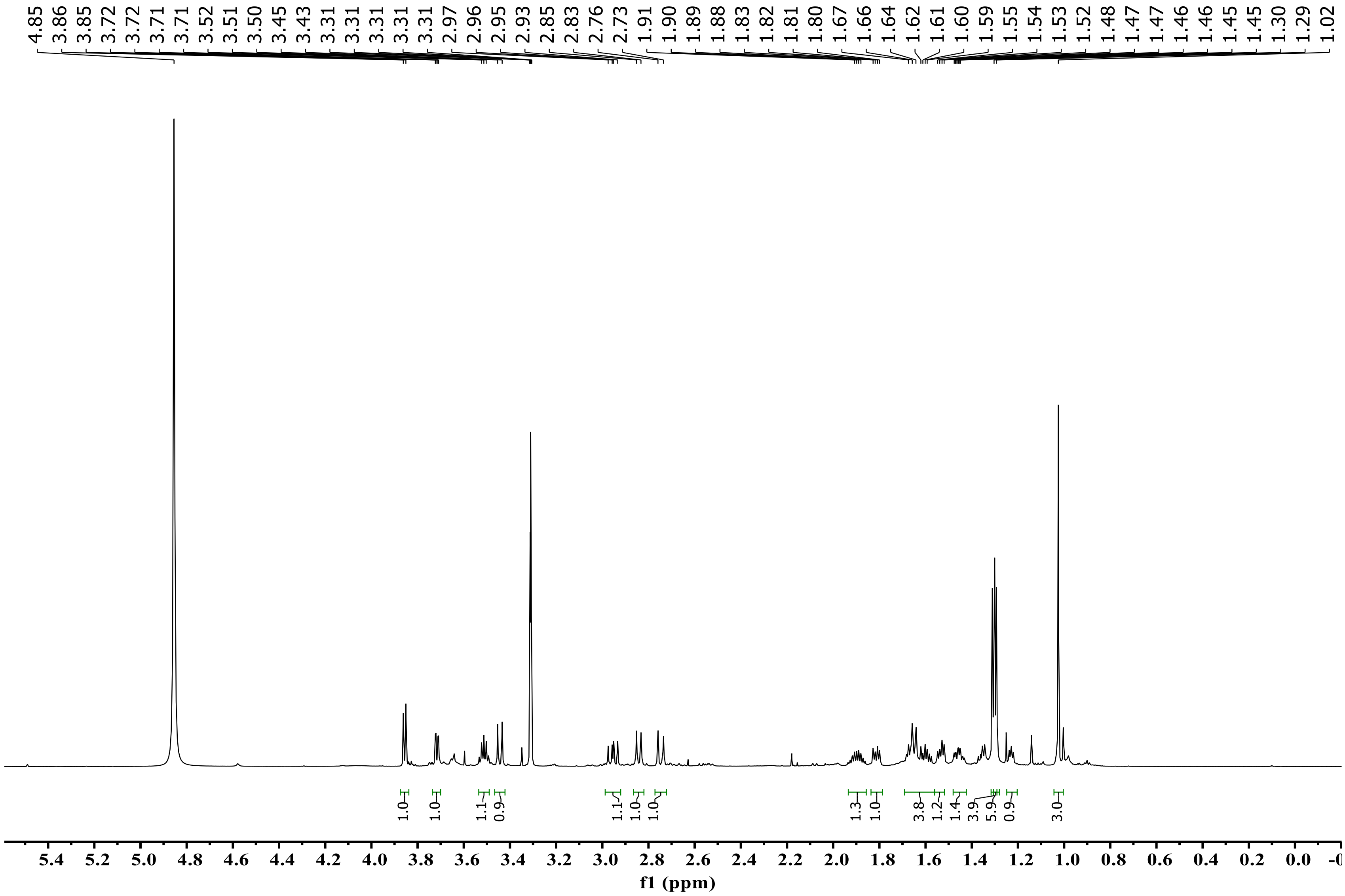

Figure S15. ^{1}H NMR (CD_3OD, 700 MHz) spectrum of **3**.

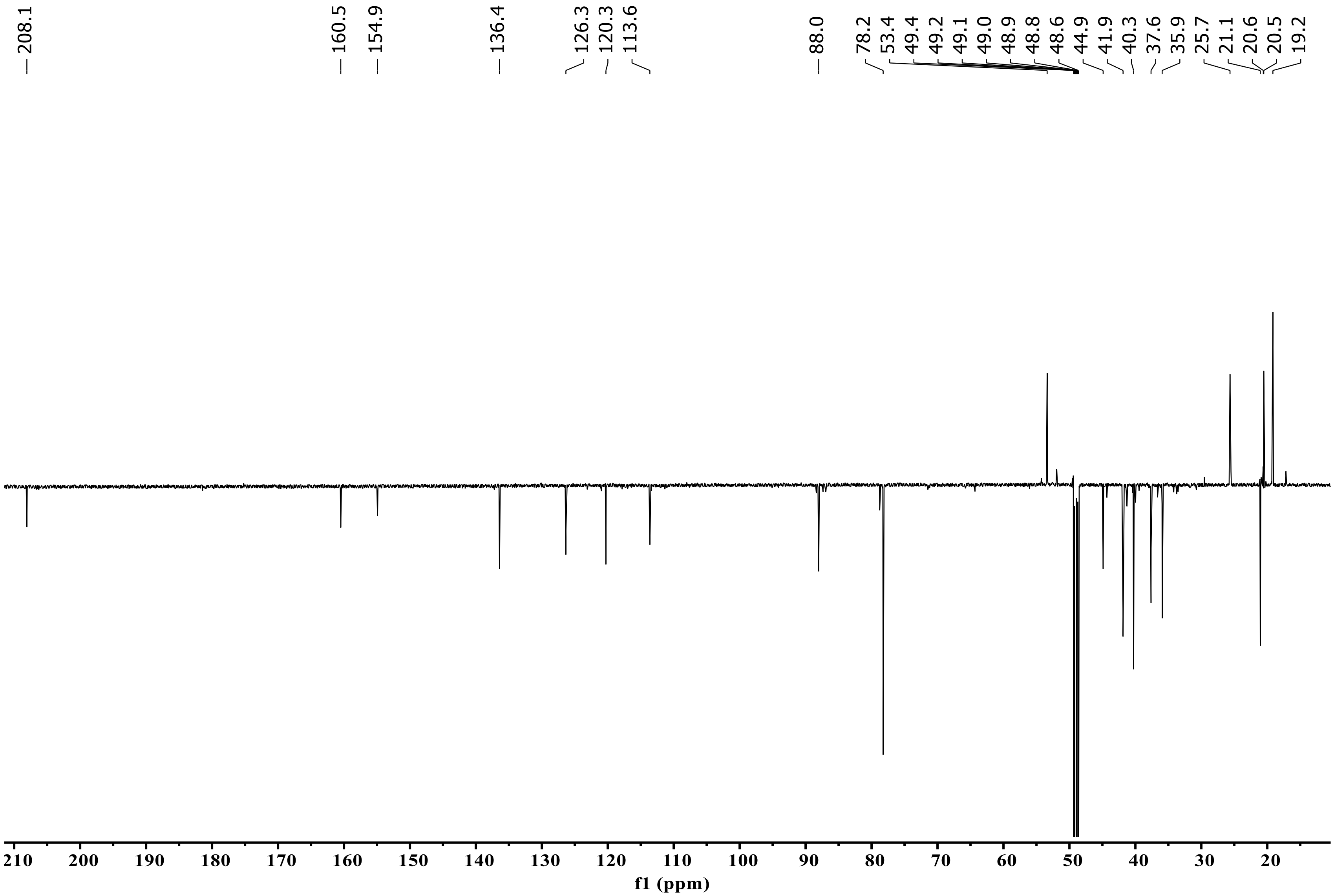

Figure S16. APT NMR (CD_3OD, 175 MHz) spectrum of **3**.

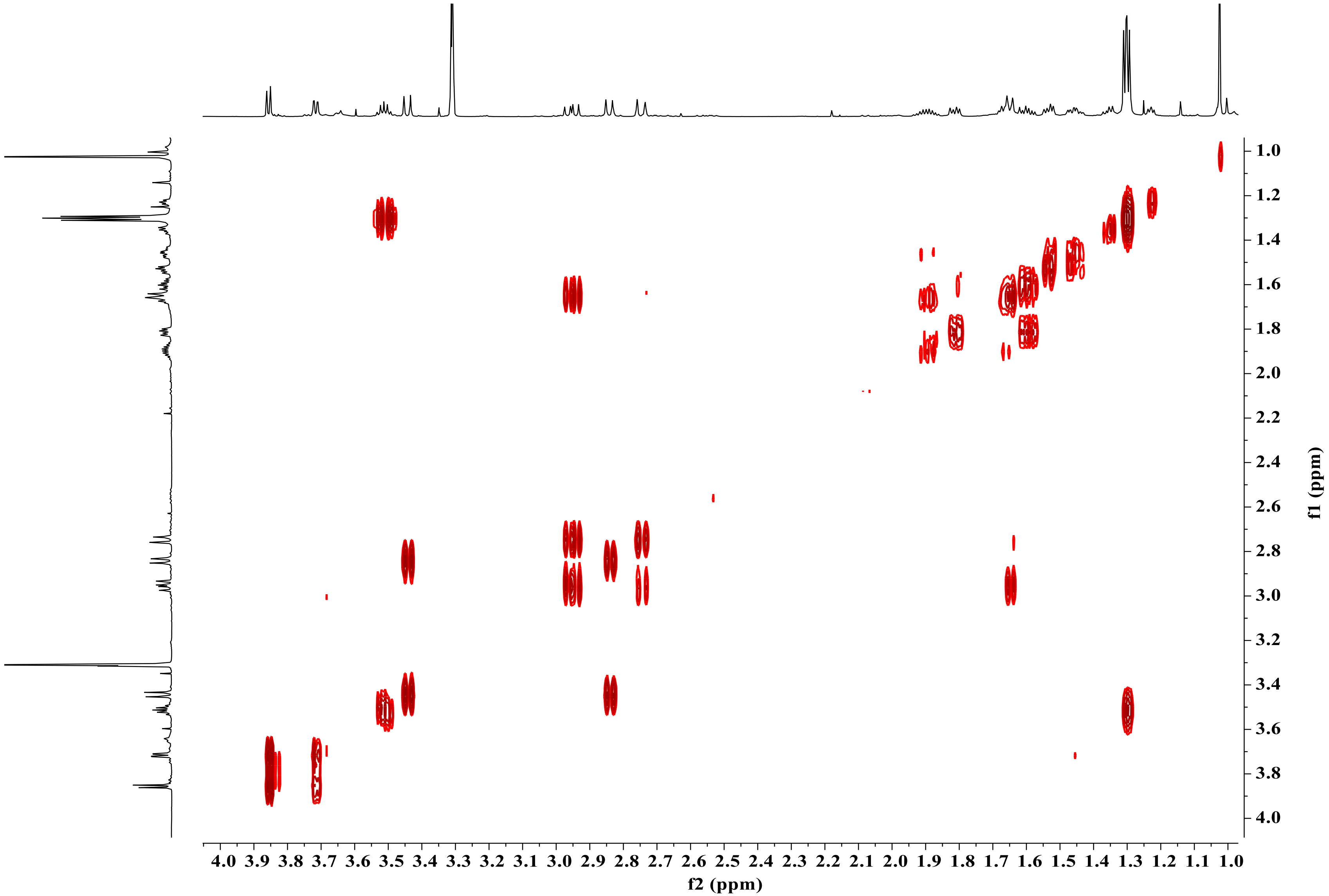

Figure S17. COSY NMR (CD_3OD, 700 MHz) spectrum of **3**.

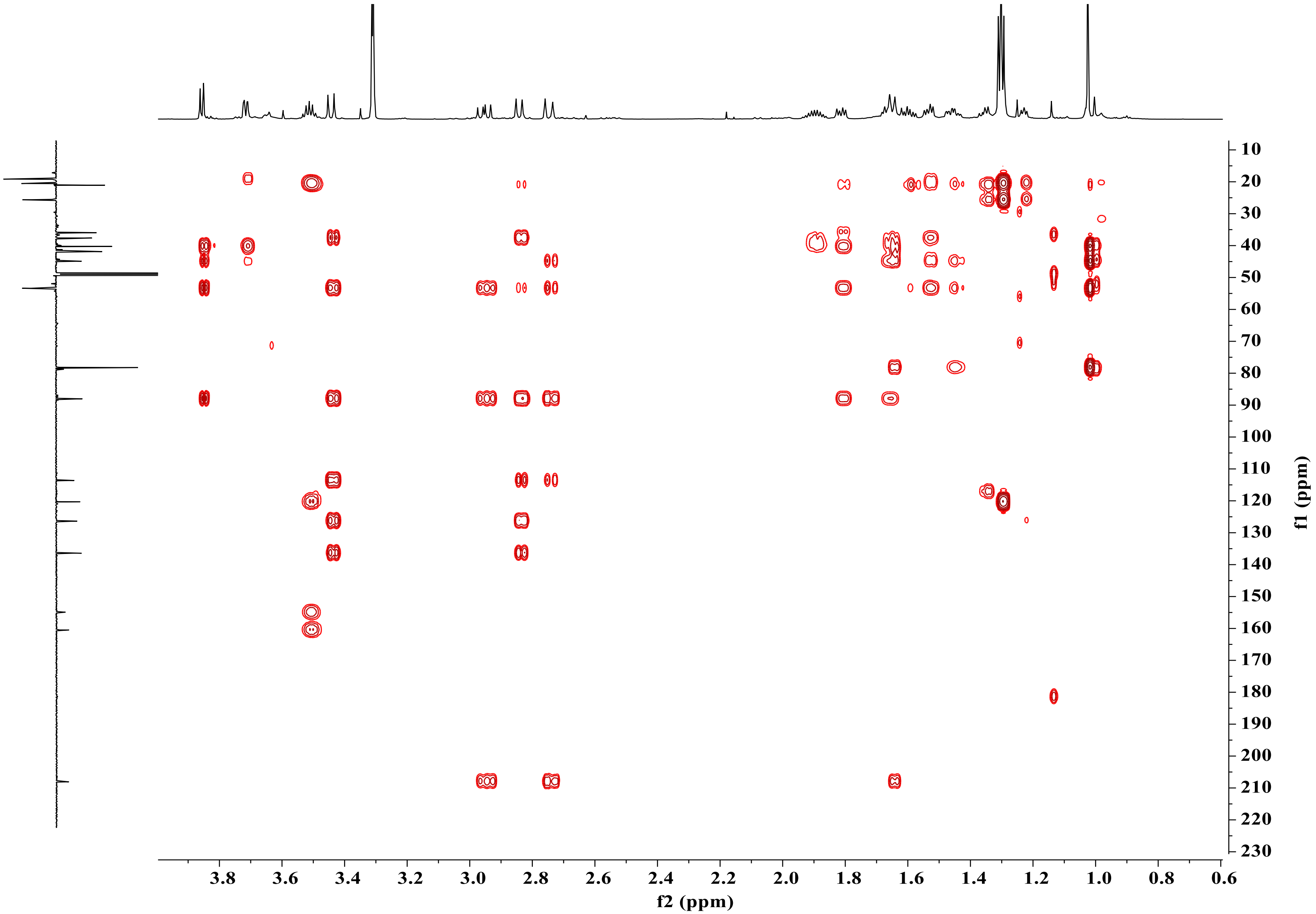

Figure S18. HMBC NMR (CD_3OD, 700 MHz) spectrum of **3**.

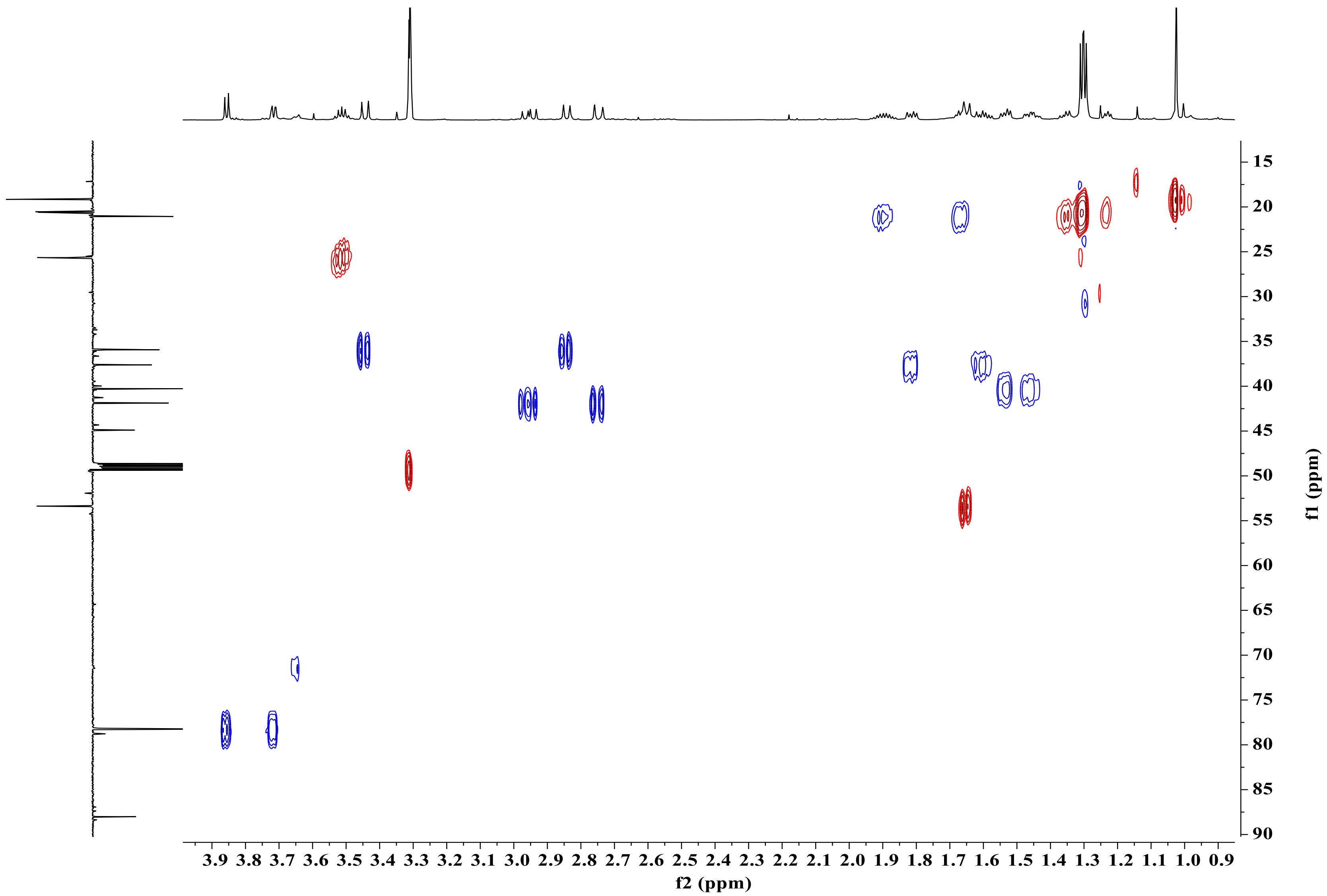

Figure S19. HSQC NMR (CD_3OD, 700 MHz) spectrum of **3**.

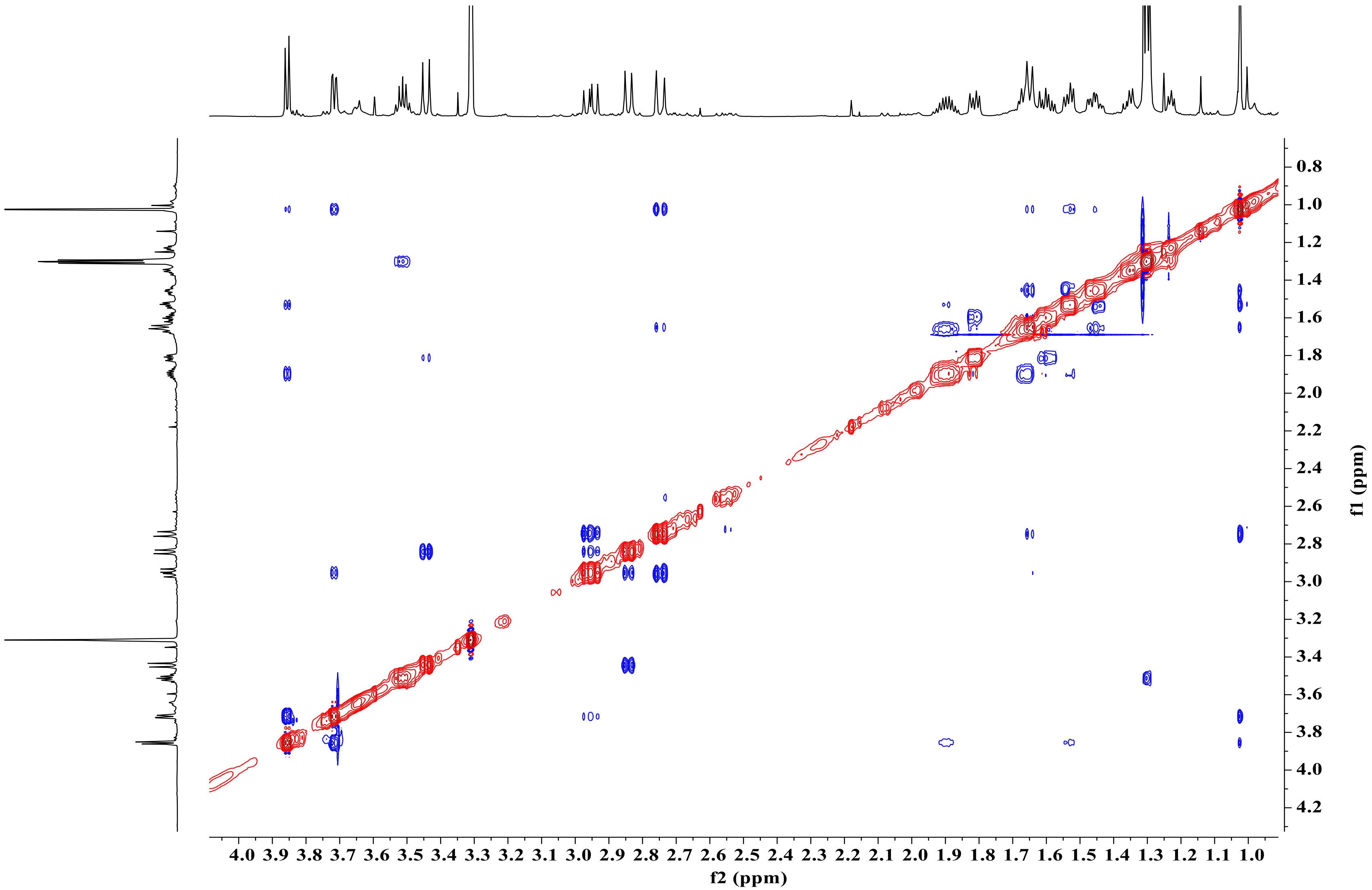

Figure S20. NOESY NMR (CD_3OD, 700 MHz) spectrum of **3**.

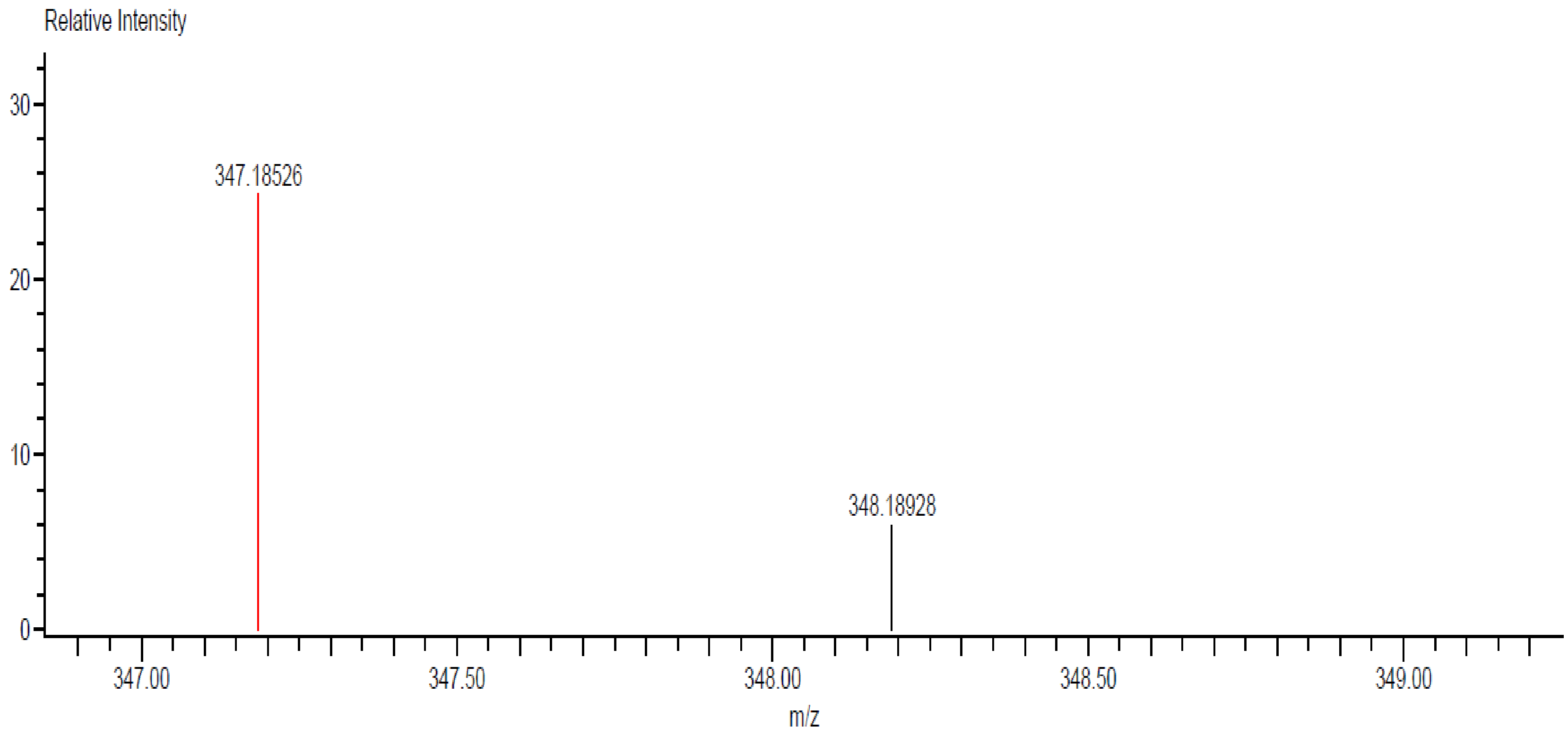

Mass	Intensity	Calc. Mass	Mass Difference (mmu)	Mass Difference (ppm)	Possible Formula	Unsaturation Number
347.18526	7713.92	347.18585	-0.59	-1.71	$^{12}C_{20}{}^{1}H_{27}{}^{16}O_5$	7.5

Figure S21. HR-DART-MS of **3**.

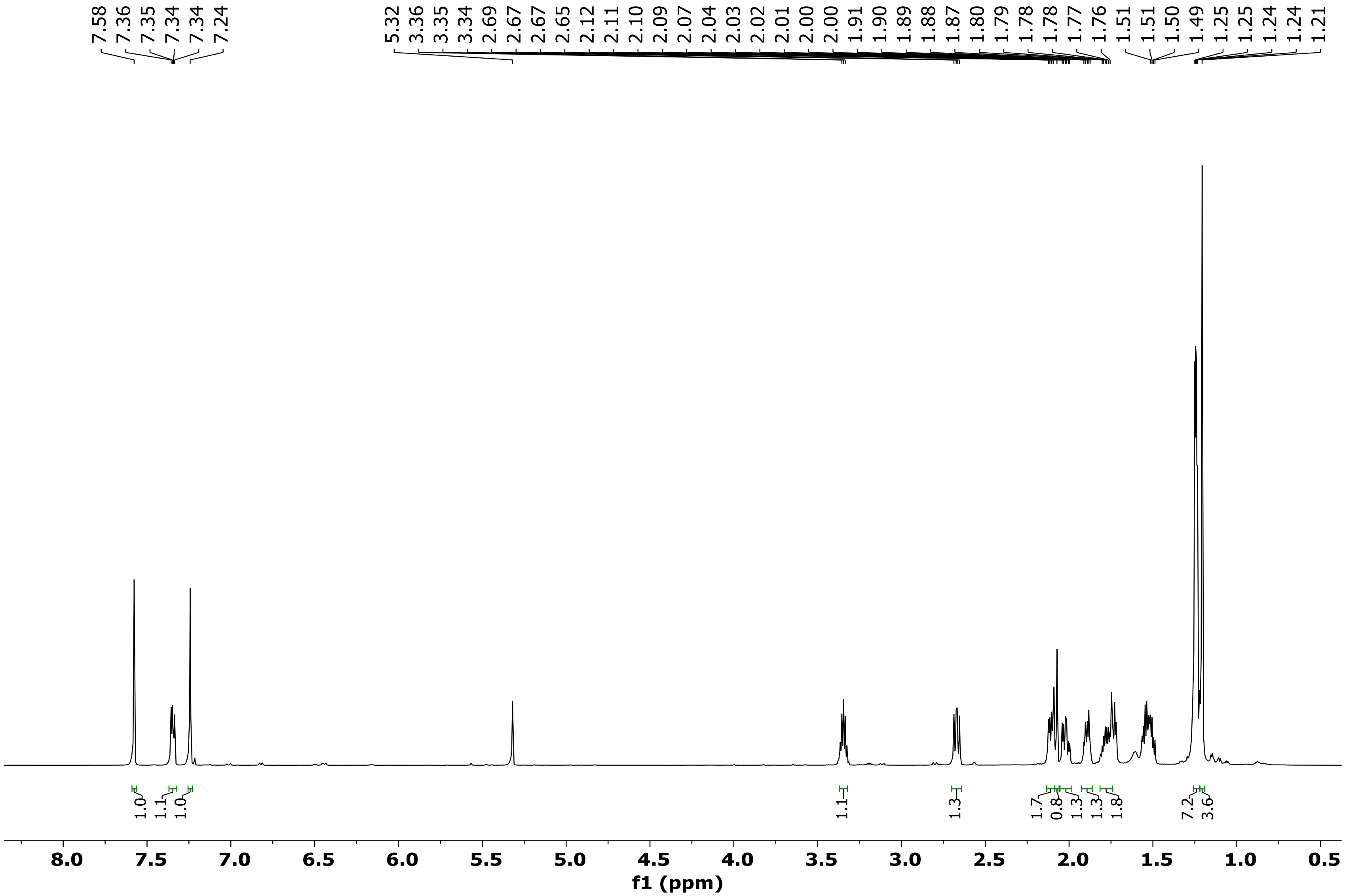

Figure S22. ^{1}H NMR (CD_2Cl_2, 700 MHz) spectrum of **5**.

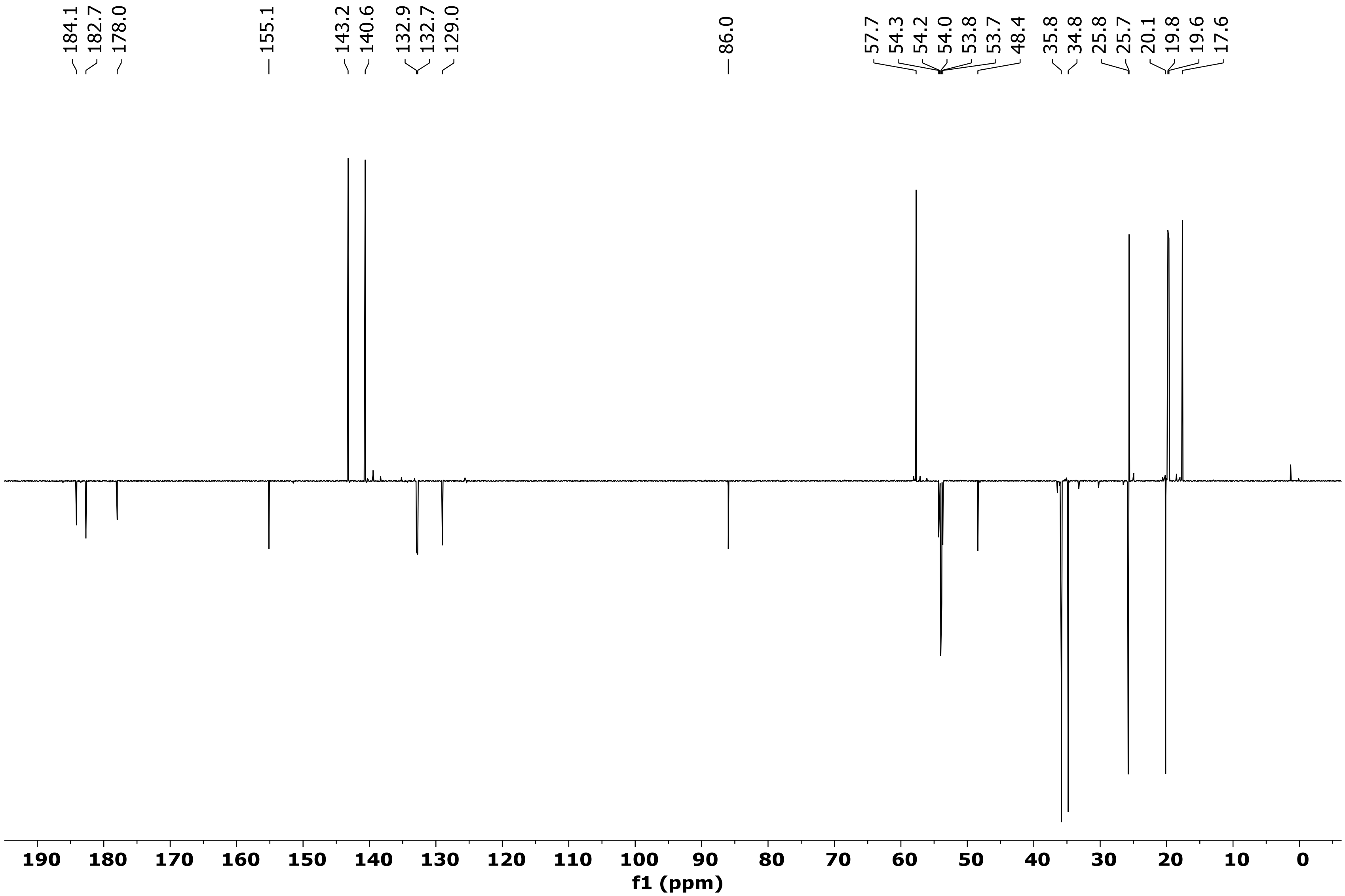

Figure S23. APT NMR (CD_2Cl_2, 175 MHz) spectrum of **5**.

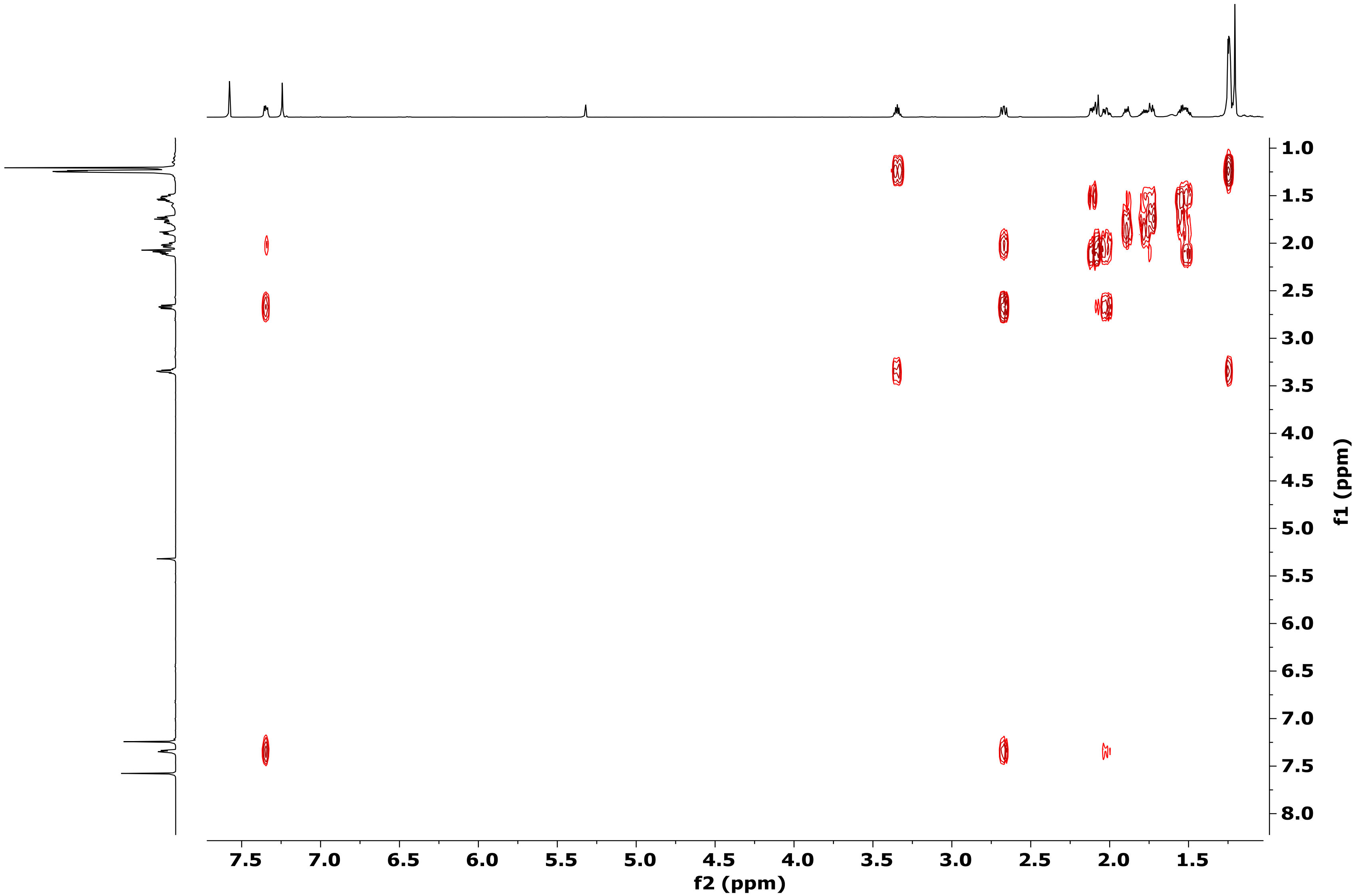

Figure S24. COSY NMR (CD_2Cl_2, 700 MHz) spectrum of **5**.

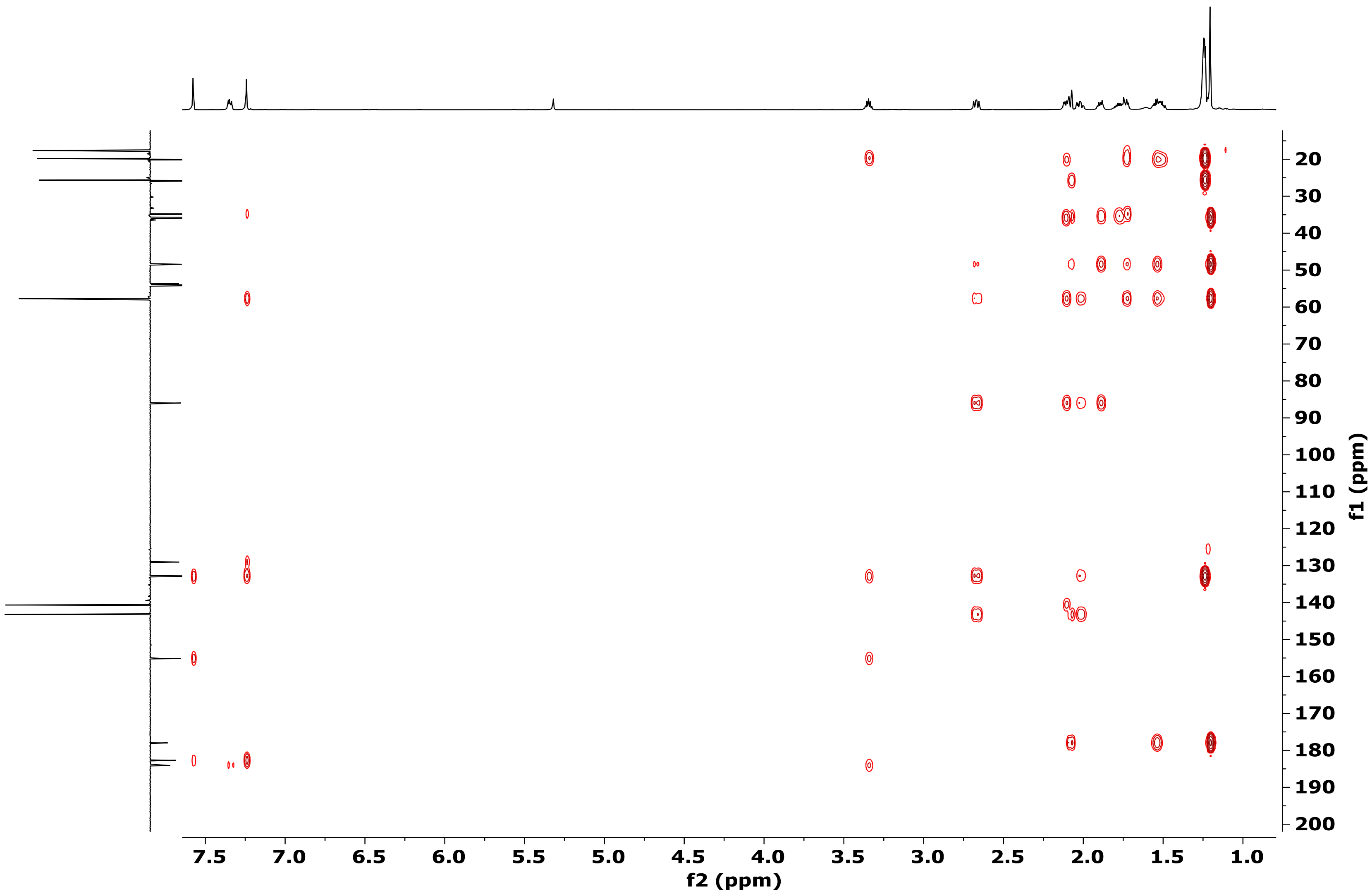

Figure S25. HMBC NMR (CD_2Cl_2, 700 MHz) spectrum of **5**.

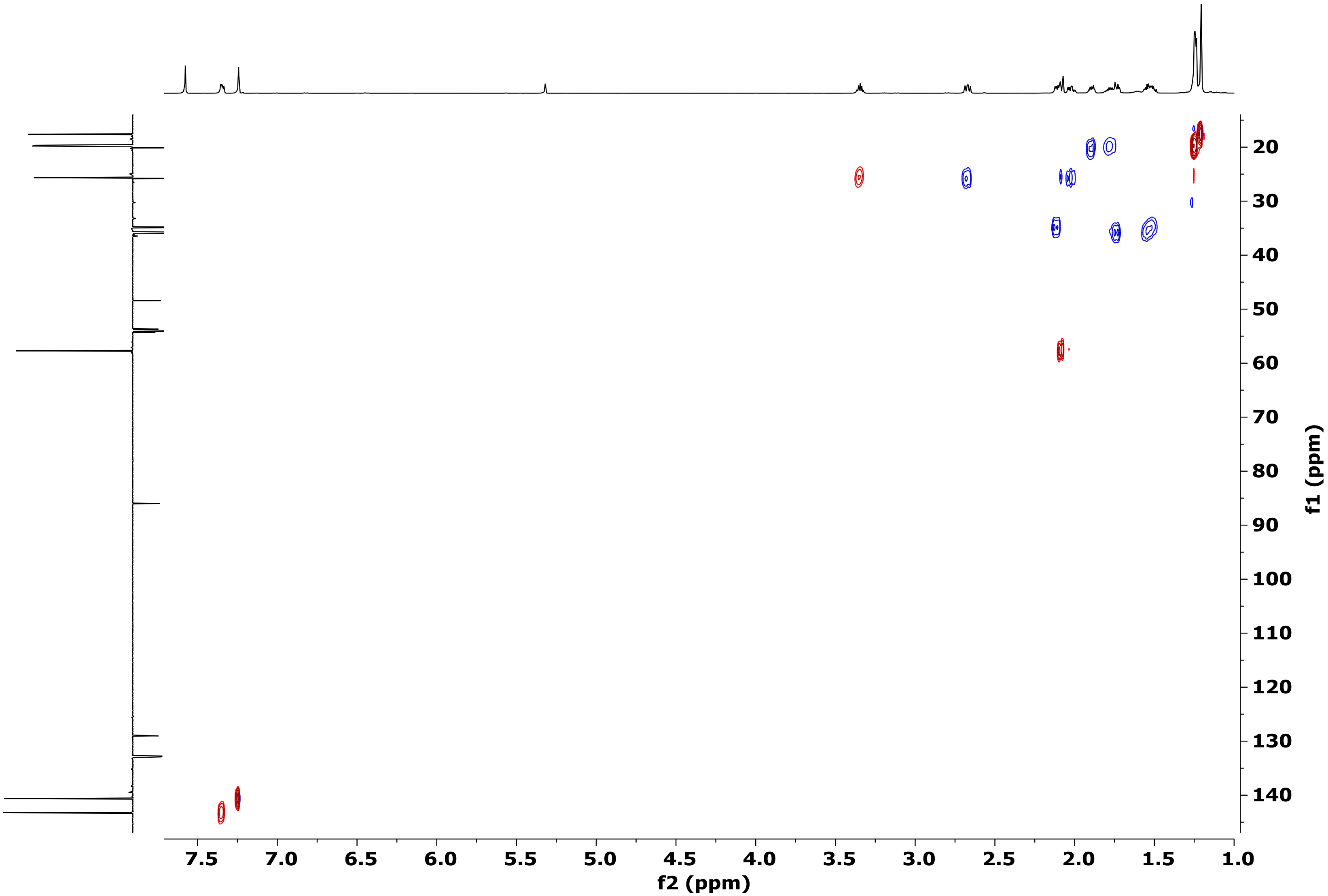

Figure S26. HSQC NMR (CD_2Cl_2, 700 MHz) spectrum of **5**.

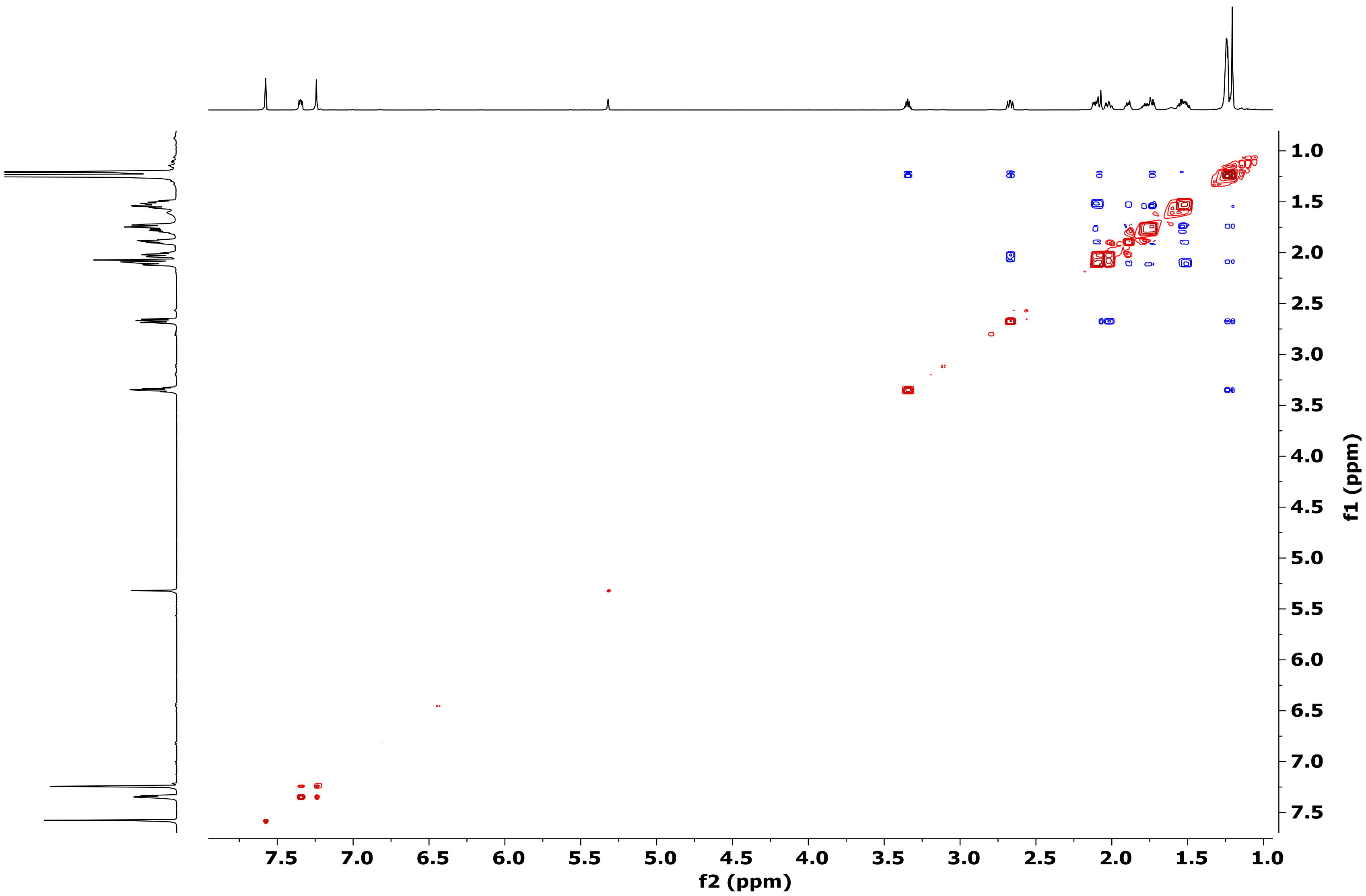

Figure S27. NOESY NMR (CD_2Cl_2, 700 MHz) spectrum of **5**.

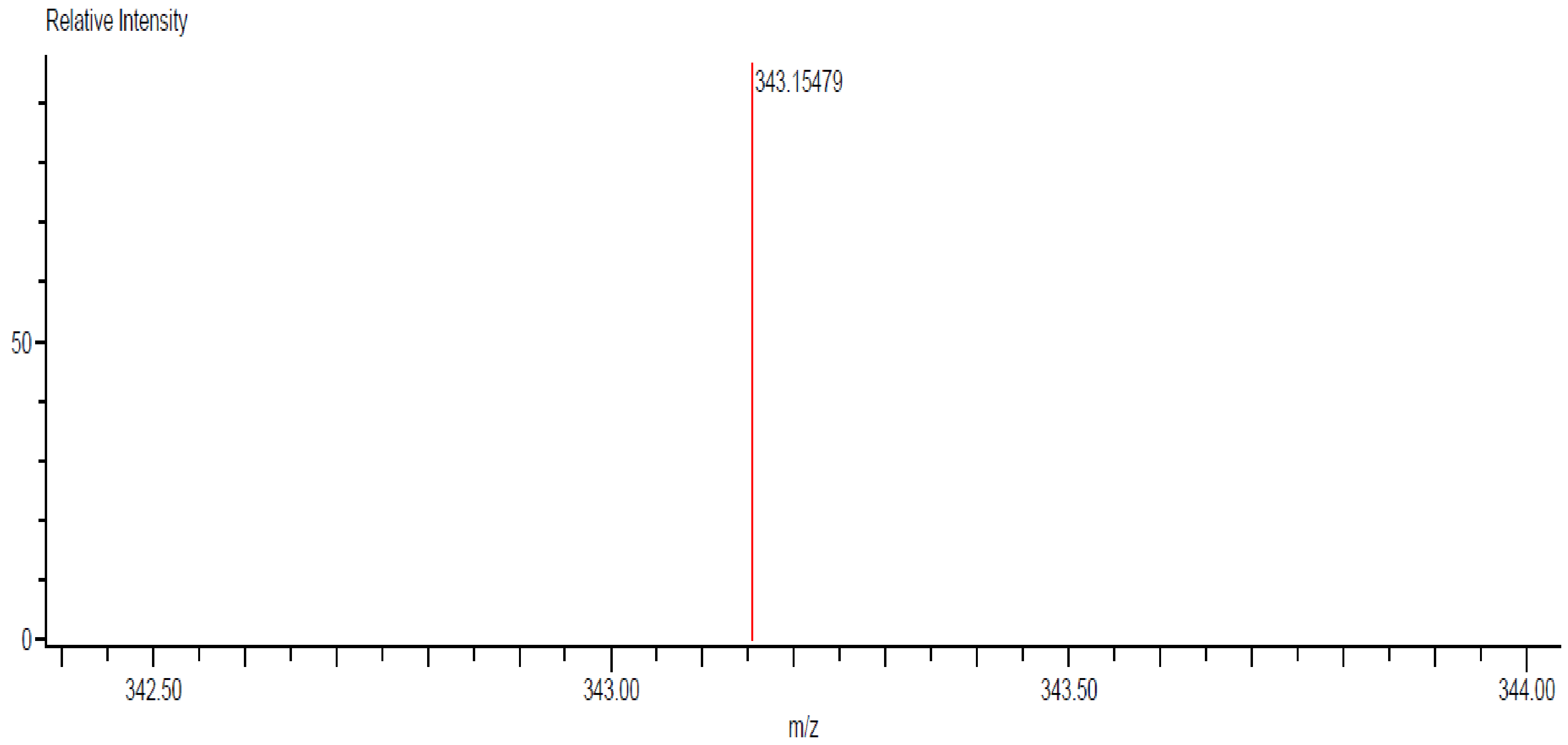

Mass	Intensity	Calc. Mass	Mass Difference (mmu)	Mass Difference (ppm)	Possible Formula	Unsaturation Number
343.15479	2866414.81	343.15455	0.25	0.72	$^{12}C_{20}{}^{1}H_{23}{}^{16}O_5$	9.5

Figure S28. HR-DART-MS of **5**.

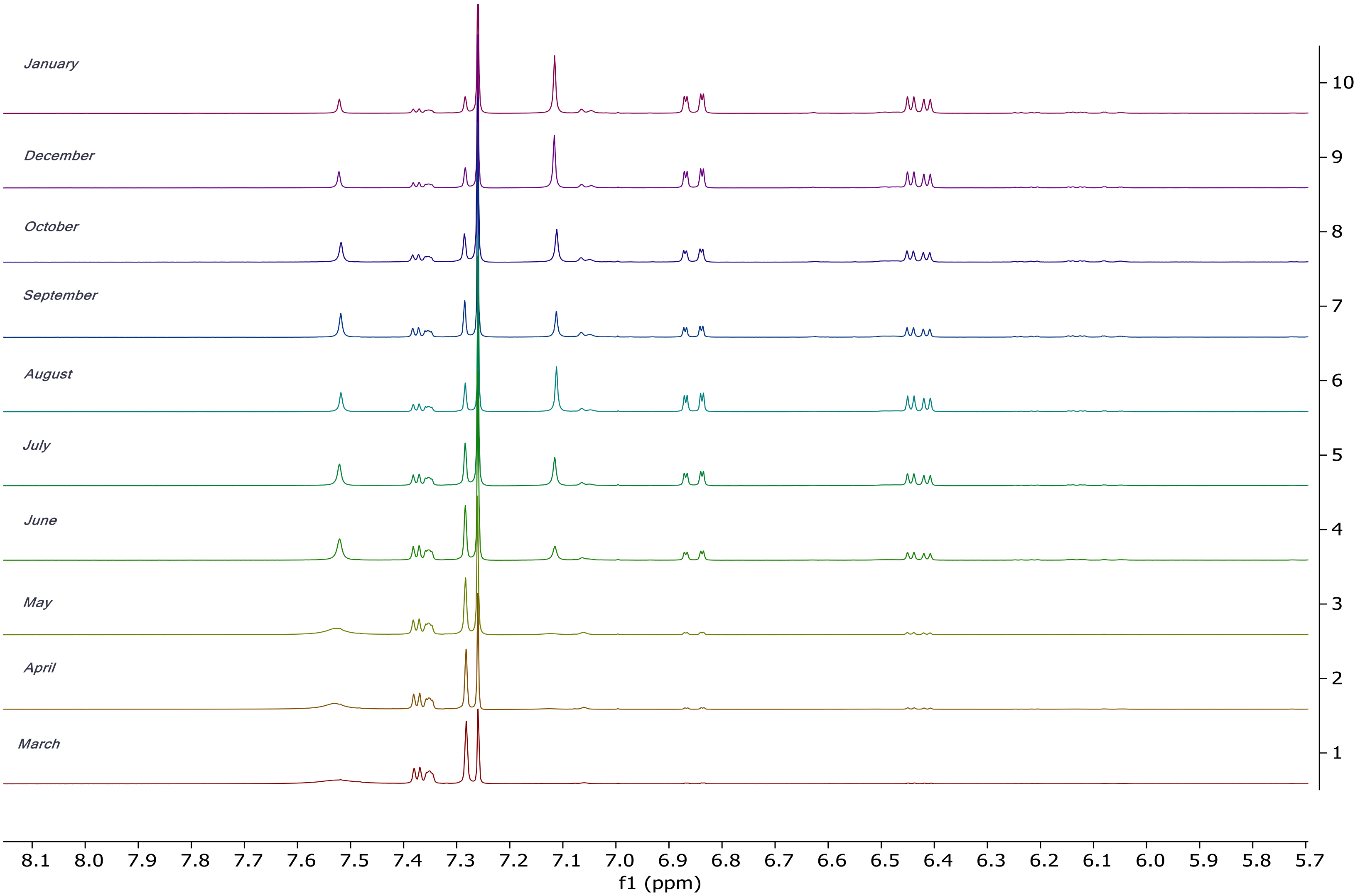

Figure S29. Isomerization from isocetexone to icetexone at room temperature.

Table S1. Primary screening of compounds **5**, **6** and **8** on antiproliferative activity at concentrations of 25 and 1.0 μM.

**Compound**	% of inhibition of cellular growth at 25 μM						
	**U251**	**PC-3**	**K562**	**HCT-15**	**MCF-7**	**SKLU-1**	**COS-7**
**5**	100	100	95.1	100	100	100	100
**6**	100	100	90.2	100	100	100	100
**8**	NC	NC	11.4	34.5	17.7	6.2	7.0
	% of inhibition of cellular growth at 1 μM						
**5**	82.9	45.7	88.6	55.6	38.3	84.4	36.1
**6**	38.6	3.0	70.3	15.8	11.8	50.3	19.1

U251 = human glioblastoma; PC-3 prostate cancer; K562 = human chronic myelogenous; leukemia; HCT-15 human colon cancer; MCF-7 = breast cancer; SKLU-1 = human lung; adenocarcinoma; COS-7 = normal monkey kidney.

Table S2. Growth inhibitory activity of compounds **5**-**7**, **9**, and **10** from *Salvia carranzae* on the root elongation and seed germination of *Amaranthus hypochondriacus*, *Trifolium pratense*, *Medicago sativa*, and *Panicum miliaceum* at concentration of 100 µg/mL.

Seed/Compound	**5**		**6**		**7**		**9**		**10**		**Rival***	
	Root Elongation	Seed germination	Root Elongation	Seed germination	Root Elongation	Seed germination	Root Elongation	Seed germination	Root Elongation	Seed germination	Root Elongation	Seed germination
Amaranthus hypochondriacus	54.82±0.05*	NA	4.07± 0.14	NA	0.185±0.128	NA	18.64±0.14*	2.6±1.41	35.54±0.11*	11.3±1.91	62.5±0.21*	5.2±1.26
Trifolium pratense	19.16±0.20*	4.3±2.8	18.86±0.18*	5.4	0.06±0.17	3.5±2.2	1.37±0.21	7.6±2.1	14.05±0.08*	NA	48.9±0.17*	5.4 ±3.10
Medicago sativa	8.68±0.21	NA	1.15±0.23	NA	14.6±0.13*	2.7 ±2.22	15.48±0.12*	NA	4.50±0.15	NA	36.6±0.23*	NA
Panicum miliaceum	14.93±0.11*	NA	2.08±0.08	NA	2.28±0.09	6.8±1.71	7.23±0.10	NA	35.20±0.11*	NA	54.2±0.24*	NA

Results were analyzed by analysis of variance (ANOVA) and Tukey statistical tests utilizing GraphPad Prism ver. 6.01 statistical computer software (GraphPad software, La Jolla, CA, USA). Data are represented as mean (n=4) ± standard deviation (SD). A P value of ≤0.05 (*) was employed to indicate statistical significance. NA = No active. * Glyphosate, N-(phosphonomethyl)glycine (200 µg/mL).
